# Immune microenvironment modulation following neoadjuvant therapy for oesophageal adenocarcinoma: a translational analysis of the DEBIOC clinical trial

**DOI:** 10.1016/j.esmoop.2024.103930

**Published:** 2024-10-11

**Authors:** E. Scanlon, A. Lavery, M. Albraikat, L. Stevenson, C. Kennedy, R. Byrne, A. Walker, B. Mullan-Young, D.T. McManus, P.S. Virdee, L. Elhussein, J. Turbitt, D. Collinson, Z. Miedzybrodzka, S. Van Schaeybroeck, S. McQuaid, J.A. James, S.G. Craig, J.K. Blayney, R.D. Petty, D.P. Harkin, R.D. Kennedy, M.M. Eatock, M.R. Middleton, A. Thomas, R.C. Turkington

**Affiliations:** 1 Patrick G Johnston Centre for Cancer Research, Queen’s University Belfast, Belfast; 2 Precision Medicine Centre of Excellence, The Patrick G. Johnston Centre for Cancer Research, Queen's University Belfast, Belfast; 3 Department of Pathology, Belfast City Hospital, Belfast Health and Social Care Trust, Belfast; 4 Centre for Statistics in Medicine, University of Oxford, Oxford; 5 Medical Genetics, School of Medicine, Medical Sciences, Nutrition and Dentistry, University of Aberdeen, Aberdeen; 6 Northern Ireland Biobank, The Patrick G. Johnston Centre for Cancer Research, Queen's University Belfast, Belfast; 7 Division of Molecular and Clinical Medicine, Ninewells Hospital and School of Medicine, University of Dundee, Dundee; 8 Almac Diagnostic Services Ltd, Craigavon; 9 Northern Ireland Cancer Centre, Belfast City Hospital, Belfast Health and Social Care Trust, Belfast; 10 Department of Oncology, University of Oxford, Oxford; 11 University of Leicester, Leicester, UK

**Keywords:** oesophageal cancer, adenocarcinoma, AZD8931, gene expression signature

## Abstract

**Background:**

The Dual Erb B Inhibition in Oesophago-gastric Cancer (DEBIOC) trial reported an acceptable safety profile for neoadjuvant oxaliplatin and capecitabine (Xelox) ± AZD8931 in oesophageal adenocarcinoma (OAC) but limited efficacy. We evaluated the impact of neoadjuvant Xelox ± AZD8931, a novel small-molecule inhibitor with equipotent activity against epidermal growth factor receptor (EGFR), human epidermal growth factor receptor (HER)2 and HER3, on biological pathways using a unique software-driven solution.

**Patients and methods:**

Transcriptomic profiles from 25 pre-treatment formalin-fixed paraffin-embedded OAC biopsies and 18 matched resection specimens, treated with Xelox + AZD8931 (*n* = 16) and Xelox alone (*n* = 9), were analysed using the Almac clara^T^ total mRNA report analysing 92 gene signatures, 100 unique single-gene drug targets and 7337 single genes across 10 hallmarks of cancer. Gene-set enrichment analysis (GSEA) was utilised to investigate pathways governing pathological response. Tumour-infiltrating lymphocytes (TILs) were assessed digitally using the QuPath software.

**Results:**

Hierarchical clustering identified three molecular subgroups classified by activation of innate immune signalling. The immune-high subgroup was associated with HER2 positivity, increased pathological response and a marked reduction in immune signalling and TILs following neoadjuvant therapy. The immune-low cluster was predominantly HER2/EGFR-negative, and EGFR positivity was associated with the immune-mixed subgroup. Treatment with neoadjuvant therapy induced common resistance mechanisms, such as angiogenesis and epithelial–mesenchymal transition signalling, and a reduction in DNA repair signatures. Addition of AZD8931 was associated with reduction of expression of EGFR, HER2 and AKT pathways and also promoted an immunosuppressive microenvironment. GSEA showed that patients with a pathological response to treatment had increased immune signalling, whereas non-responders to neoadjuvant therapy were enriched for nucleotide repair and cellular growth through the action of E2F transcription factors.

**Conclusion:**

OAC may be subdivided into three immune-related subgroups which undergo modulation in response to neoadjuvant therapy with marked suppression of the immune microenvironment in HER2-positive/immune-high tumours.

## Highlights

• We identified three subgroups in OAC classified by innate immune signalling: immune-high, immune-low and immune-mixed.
• The immune-high subgroup is associated with HER2 positivity, pathological response and changes in immune signalling.
• The immune-mixed cluster is associated with EGFR positivity; the immune-low cluster is associated with HER2/EGFR negativity.
• Loss of immune signalling in the immune-high cluster is associated with a reduction in TILs.
• Treatment with the pan-HER inhibitor AZD8931 may contribute to an immunosuppressive phenotype.

## Introduction

Oesophageal adenocarcinoma (OAC) is a markedly heterogeneous cancer characterised by high mutation rates, chromosomal instability and amplification of receptor tyrosine kinases (RTKs).^1^, ^2^, ^3^, ^4^ The incidence of OAC has risen rapidly in the Western world, with the highest rates occurring in the UK where 5-year survival remains poor at 17%.^5^, ^6^, ^7^ Approximately two-thirds of patients are diagnosed at an advanced or metastatic stage and limited response rates to currently available treatment regimens contribute to poor survival outcomes. Even when surgical resection is possible, neoadjuvant chemotherapy provides only limited benefit, with 17%-22% of patients showing a pathological response to treatment, highlighting the need to improve the efficacy of neoadjuvant therapy for all OAC patients.^8^^,^^9^

The ErbB family of RTKs are implicated in multiple cancer types via dysregulation of pathways involved in oncogenic processes such as epithelial–mesenchymal transition (EMT), tumour invasion and cell migration.^10^ There are four members of the ErbB RTK family, namely ErbB1 [epidermal growth factor receptor (EGFR)/human epidermal growth factor receptor (HER1)], ErbB2 (HER2), ErbB3 (HER3) and ErbB4 (HER4). EGFR and HER2 have long been implicated in tumour progression, and development of targeted therapies towards these components of the ErbB signalling cascade has been extensively explored in multiple tumour types, including OAC.^11^ In advanced gastric or gastro-oesophageal junction cancer, the ToGA trial demonstrated a statistically significant improvement in overall survival (OS) from 11.1 to 13.4 months with the addition of trastuzumab, a monoclonal antibody targeted to HER2, to standard cisplatin-based chemotherapy.^12^ Inhibition of EGFR has also shown some activity in the second-line setting for oesophageal cancer where, despite there being no improvement in OS with gefitinib versus placebo in an intention-to-treat population, a subgroup of EGFR copy number gain tumours (20.1%) demonstrated improved OS (hazard ratio 0.59, 95% confidence interval 0.35-1.00, *P* = 0.05).^13^^,^^14^ Contrastingly, combined inhibition of EGFR and HER2 has shown limited efficacy in the advanced gastro-oesophageal cancer population.^15^^,^^16^

The phase I Dual ErbB Inhibition in Oesophago-gastric Cancer (DEBIOC) clinical trial assessed the efficacy of a small-molecule inhibitor with equipotent activity to EGFR, HER2 and HER3 (AZD8931) in combination with Xelox.^17^^,^^18^ The trial sought to establish a maximum tolerated dose of AZD8931 in combination with Xelox in patients with advanced oesophago-gastric cancer followed by a dose-expansion phase of Xelox plus AZD8931 compared to Xelox alone as neoadjuvant therapy in surgically resectable OAC patients. A recommended phase II dosage of 20 mg twice daily in a 4-days-on/3-days-off per week schedule had an acceptable safety profile, but limited evidence of improved efficacy.

We hypothesised that transcriptional analysis of DEBIOC patient biopsy and resection specimens could (i) provide insight into the molecular stratification of OAC, (ii) indicate how molecular subtypes may alter in response to neoadjuvant therapy and (iii) elucidate potential mechanisms of resistance to EGFR and HER2 inhibition in OAC. We applied a unique, software-driven solution to classify biologically relevant gene expression signatures (clara^T^, Almac Diagnostic Services) to further understand transcriptional changes occurring in each treatment arm of the clinical trial and the sample cohort as a whole, while relating these changes to the hallmarks of cancer.

## Patients and methods

### Patient cohort

All patients included in this study were enrolled in the randomised phase II dose-expansion component of the DEBIOC clinical trial (EudraCT 2011-003169-13, ISRCTN-68093791, ethics no. 12/SC/0090) as previously described (Supplementary Figure S1, available at https://doi.org/10.1016/j.esmoop.2024.103930).^17^ Patients were randomised 2 : 1 to receive either oxaliplatin (130 mg/m^2^ i.v. over 2 h on day 1 of every cycle) and capecitabine (1250 mg/m^2^ bd) (Xelox) + AZD8931 (20 mg bd 4 days on/3 days off) for two 21-day cycles, or Xelox alone as neoadjuvant treatment before oesophagectomy at four UK centres (Belfast, Leicester, Oxford and Bristol). Pathological response was assessed in the matched resection specimens according to the method described by Mandard et al. with a responder defined as tumour regression grade (TRG) ≤2.^19^ Samples which passed histopathology quality control were taken forward for analysis as outlined in the CONSORT diagram (Supplementary Figure S2, available at https://doi.org/10.1016/j.esmoop.2024.103930).

The DEBIOC study was conducted in accordance with the International Conference of Harmonisation of Good Clinical Practice and the Declaration of Helsinki. Ethical approval was provided by the NRES Committee South Central (12/SC/0090). All patients provided written informed consent for participation.

### Transcriptomic profiling using the Almac Diagnostics’ clara^T^ total mRNA report

Transcriptomic profiling of 25 pre-treatment formalin-fixed paraffin-embedded (FFPE) OAC endoscopic biopsies and 18 surgical resection specimens was carried out using the Almac Diagnostics Xcel™ array (Almac, Craigavon, UK) as previously described.^20^ Samples which passed quality control were processed using the clara^T^ total mRNA report (Almac Diagnostic Services, version 3.0.0). Clara^T^ provides a comprehensive overview of tumour profiles using 92 gene signatures, 100 unique single-gene drug targets (SGDTs) and 7337 single genes across the 10 hallmarks of cancer (Supplementary Figure S3, available at https://doi.org/10.1016/j.esmoop.2024.103930). A pre-defined threshold of 0.3403 was used to define DNA damage immune response (DDIR) signature status, whereby a score >0.3403 was classified as DDIR positive and ≤0.3403 as DDIR negative.^20^ Receiver operating characteristic (ROC) curves were constructed for each of the 92 reported signatures with an area under the ROC curve (AUC) >0.65 determining signatures predictive of progression-free survival (PFS) outcomes at 24 months.

### Semi-supervised clustering

Utilising clara^T^ signatures within each hallmark, we carried out semi-supervised clustering of hallmark signatures in the R statistical package with ‘ConsensusClusterPlus’.^21^ Resulting clusters were filtered to those which had >70% sample consensus to a cluster within a given k ranging from 2 to 6 and were tested for clinical associations of interest using Fisher’s exact testing with Benjamini–Hochberg correction for multiple testing (for further details see the Supplementary Methods, available at https://doi.org/10.1016/j.esmoop.2024.103930).

### Immunohistochemical staining

HER2 expression was detected using the PATHWAY anti-HER2/neu (4B5) antibody on an automated immuno-stainer (Benchmark® XT, Ventana Medical Systems Inc., Tucson, AZ), according to the manufacturer’s instructions.

### EGFR FISH

EGFR FISH was carried out and scored using an established protocol in NHS Grampian.^22^ EGFR copy number gain was classified using a 6-point scale; tumours scoring 5 (high polysomy) or 6 (amplification) were classified as EGFR FISH positive; tumours scoring 1-4 were defined as EGFR FISH negative.^22^

### Identification and quantification of tumour-infiltrating lymphocytes (TILs)

Digital assessment of TILs was carried out using digitally scanned haematoxylin and eosin (H&E) whole-slide images from matched pre-treatment biopsies and surgical resection specimens. Cells were classified as ‘immune cell’ or ‘non-immune cell’ using handcrafted features within QuPath, an open-source image analysis software.^23^ The selection and assessment of regions of interest adhered to the established guidelines and were reviewed before immune analysis by a consultant pathologist (JAJ).^24^

## Results

### Patient cohort

The randomised phase II dose-expansion component of the DEBIOC study recruited 30 patients with histologically confirmed adenocarcinoma of the oesophagus or gastro-oesophageal junction treated with neoadjuvant therapy (*n* = 20 Xelox + AZD8931, *n* = 10 Xelox alone) followed by surgical resection. Samples were obtained for 26 patients of whom 3 did not proceed to surgery, 2 biopsy samples and 1 resection specimen had insufficient tumour content for analysis, 2 patients obtained a complete pathological response to neoadjuvant therapy and 2 resection specimens could not be retrieved (Supplementary Figure S2, available at https://doi.org/10.1016/j.esmoop.2024.103930, CONSORT diagram). Transcriptional profiling was successfully carried out for 25 pre-treatment FFPE endoscopic biopsies (including one duplicate biopsy) and 18 resection specimens, resulting in a total of 6 paired biopsy–resection specimens for Xelox-only-treated patients, 11 paired biopsy–resection specimens for Xelox + AZD8931-treated patients, 1 unpaired resection specimen and 8 unpaired biopsies available for analysis. There were no significant differences in the clinicopathological characteristics between the two treatment cohorts (Supplementary Table S1, available at https://doi.org/10.1016/j.esmoop.2024.103930). Two patients were classified as HER2 positive [immunohistochemistry (IHC) 3+] on their diagnostic biopsies, with one case becoming negative on the subsequent resection specimen, and two cases were HER2 negative on biopsy but HER2 positive on their resection specimens, illustrating the marked heterogeneity of HER2 expression and potential sampling error. EGFR FISH revealed amplification and high polysomy of EGFR in two and seven biopsies, respectively (Supplementary Table S2, available at https://doi.org/10.1016/j.esmoop.2024.103930).

### Hierarchical clustering of OAC biopsy samples utilising gene signatures reveals three immune biology clusters

To provide a comprehensive overview of the underlying molecular biology at diagnosis, 92 gene expression signatures, 93 SGDTs and 7337 single genes were investigated in all biopsy samples using the Almac clara^T^ assay. Semi-supervised hierarchical clustering, combined with ROC analysis, was applied to determine whether OAC biopsy samples could be stratified into subgroups reflecting prominent biological pathways and prognosis.

**Figure 1:**
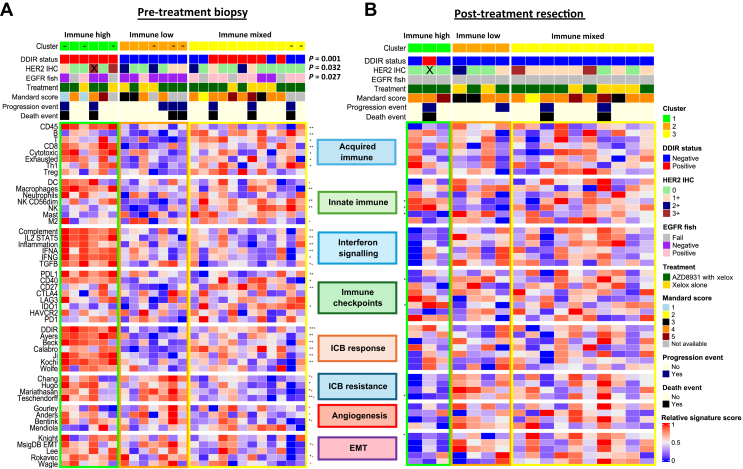
**Immune signalling, angiogenesis and EMT in biopsy and resection specimens in the DEBIOC study.** Semi-supervised clustering of (A) pre-treatment biopsies (*n* = 25) and cluster comparison where possible of their respective (B) paired resection specimens (*n* = 17). claraT signatures are grouped into gene signatures predicting acquired immunity (panel 1), innate immunity (panel 2), interferon signalling (panel 3), expression of immune checkpoint genes (panel 4), gene signatures reported to predict response to ICB (panel 5), gene signatures reported to predict resistance to ICB (panel 6), gene signatures associated with increased angiogenic signalling or response to anti-angiogenic agents (panel 7) and gene signatures associated with increased EMT signalling (panel 8). DDIR, HER2 immunohistochemistry and EGFR FISH status were shown to differ significantly between biopsy clusters (Fisher’s exact testing; *P* = 0.001, *P* = 0.032 and *P* = 0.027, respectively) and are indicated on the *x*-axis as well as treatment with either Xelox alone or Xelox + AZD8931. Pathological response, progression and death events are also highlighted. ∗*P* < 0.05; ∗∗*P* < 0.01; ∗∗∗*P* < 0.001, Kruskal–Wallis testing amongst clusters. + Indicates a signature previously identified as having an AUC score >0.65 from ROC-AUC analysis, indicating that signature is predictive of PFS outcomes at 24 months. – Indicates a biopsy specimen where no paired resection specimen is available for comparison post-treatment, all other matched samples are in the same order across biopsy and resection clusters for ease of comparison pre- and post-treatment. X indicates the HER2+ paired samples used in the digital pathology analysis. AUC, area under the ROC curve; DDIR, DNA damage immune response; EGFR, epidermal growth factor receptor; EMT, epithelial–mesenchymal transition; HER2, human epidermal growth factor receptor 2; ICB, immune checkpoint blockade; PFS, progression-free survival; ROC, receiver operating characteristic.

Clustering of the biopsy samples resulted in three distinct OAC biopsy molecular subgroups defined according to their DDIR assay status as immune-high, immune-low and immune-mixed (Figure 1A and Supplementary Figure S4, available at https://doi.org/10.1016/j.esmoop.2024.103930). We have previously shown the DDIR assay to be predictive of benefit from DNA-damaging neoadjuvant chemotherapy followed by surgical resection in OAC as well as indicative of a pro-inflammatory microenvironment.^20^^,^^25^ Biologically, the 44-gene DDIR assay indicates constitutive activation of the cyclic GMP-AMP synthase (cGAS)/stimulator of interferon genes (STING) pathway in response to endogenous DNA damage and includes immune checkpoint targets, such as programmed death-ligand 1 (PD-L1) and indoleamine 2,3-dioxygenase 1 (IDO-1), as well as several inflammatory cytokines.^26^ A significant association was observed between HER2 IHC and the three immune clusters (Fisher’s exact test, *P* = 0.03) with both HER2-positive samples (HER2 IHC score 3+) found within the immune-high cluster, which also contained up-regulation of signatures representative of both response and resistance to immune checkpoint blockade (ICB) as well as elevated angiogenesis and EMT signalling. Analysis of single-gene immune checkpoints showed increased levels of PD-L1 (*P* = 0.009) and CD40 (*P* = 0.031) expression in the immune-high cluster compared to remaining clusters as well as signatures associated with specific immune cell types, such as macrophages (*P* = 0.006), CD8+ T cells (*P* = 0.012) and interferon α and γ signalling (*P* = 0.004 and *P* = 0.012, respectively) (Supplementary Table S3, available at https://doi.org/10.1016/j.esmoop.2024.103930). We also noted a significant association of EGFR FISH classification with the three immune clusters (Fisher’s exact test, *P* = 0.0202) with EGFR FISH positivity primarily aligned with the immune-mixed cluster and all but one of the immune-low cluster being EGFR FISH negative.

Following ROC curve analysis, 11 signatures resulted in AUC scores of >0.65, indicative of predicting PFS outcomes in our patient cohort based on whether or not relapse had occurred at 24 months (Supplementary Table S4, available at https://doi.org/10.1016/j.esmoop.2024.103930). Five of the 11 signatures (45.5%), including the top two ranked signatures, were related to inflammation/immune-oncology hallmarks, indicating a potential role of immune signalling in OAC patient survival.

### Neoadjuvant therapy alters immune, angiogenesis and EMT signalling pathways

To investigate alterations in the immune clusters and molecular signalling during neoadjuvant therapy, we compared signature scores and semi-supervised consensus clustering of biopsies with their matched resection specimens (Figure 1B and Supplementary Table S5, available at https://doi.org/10.1016/j.esmoop.2024.103930). There was a distinct loss of immune signalling after treatment in patients in the immune-high cluster resulting in a reduction of expression in signatures associated with resistance to ICB (Hugo et al.,^27^ *P* = 0.048; Teschendorff et al.,^28^ *P* = 0.024), angiogenesis (Bentink et al.,^29^ *P* = 0.024) and EMT (MSigDB EMT, *P* = 0.048; Lee et al.,^30^ *P* = 0.048). Decreased scores were also observed in immune checkpoints, such as CD40 (*P* = 0.024), with an increase in IDO-1 (*P* = 0.024) and natural killer (NK) cell signalling (*P* = 0.024). No significant changes were observed in the immune-mixed or immune-low clusters (Supplementary Tables S6-S8, available at https://doi.org/10.1016/j.esmoop.2024.103930).

**Figure 2:**
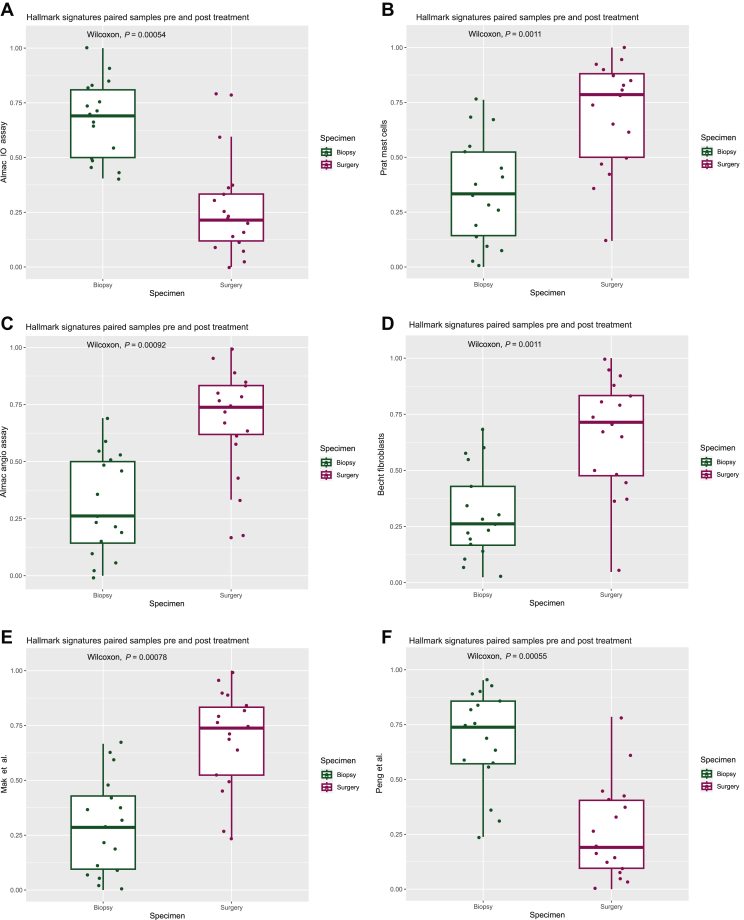

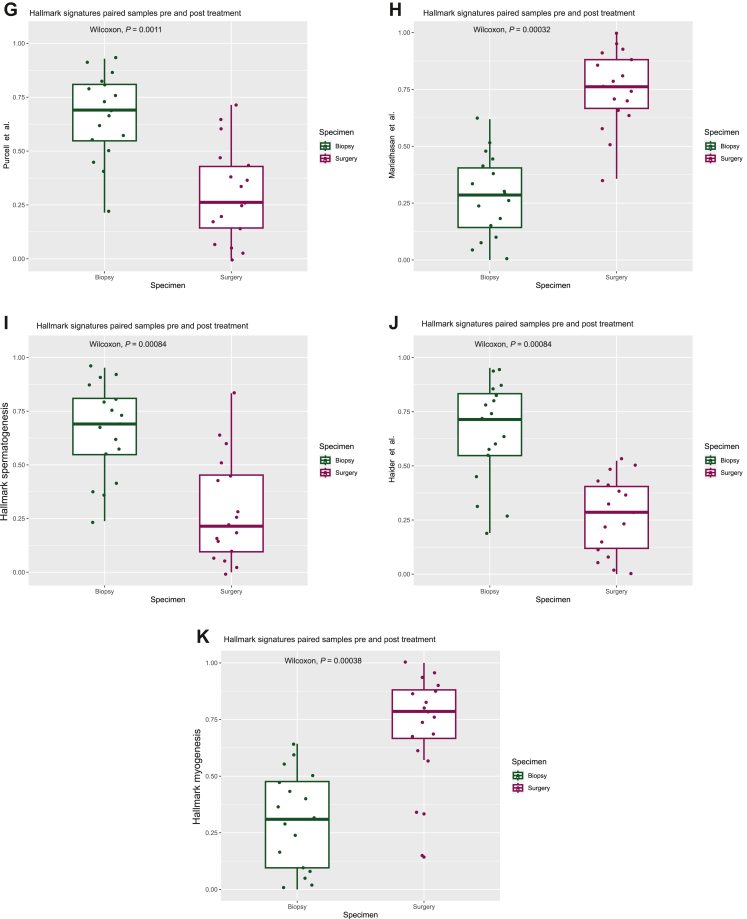

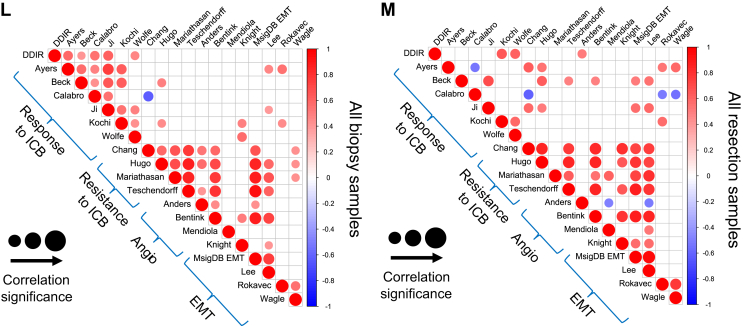
**Neoadjuvant therapy alters immune, angiogenesis and EMT signalling pathways.** Boxplots comparing signature scores (percentile ranks) using Wilcoxon signed rank testing between paired biopsies (*n* = 17) and resection specimens demonstrated that neoadjuvant therapy induced significant changes in signatures related to (A) DDIR score (annotated as Almac IO assay, *P* < 0.001), (B) mast cells (*P* = 0.001), (C) angiogenesis (*P* < 0.001), (D and E) EMT (*P* = 0.001 and *P* < 0.001 respectively), (F) genomic instability (*P* < 0.001), (G) senescence (*P* = 0.001), (H) TGF-β signalling (*P* < 0.001), (I and J) metabolism (both *P* < 0.001) and (K) myogenesis (*P* < 0.001). (L and M) Plots displaying significant results only from Spearman’s rank correlation testing (*P* < 0.05) of gene signatures relating to response/resistance to ICB, angiogenic or EMT signalling in biopsy (*n* = 25) (L) and resection (*n* = 17) (M) specimens. Increasing significance and strength of correlations are indicated by an increase in size and colour intensity of circles on the heatmap where red indicates positive correlation and blue indicates negative correlation. DDIR, DNA damage immune response; EMT, epithelial–mesenchymal transition; ICB, immune checkpoint blockade; TGF, transforming growth factor.

Comparing signature scores before and after neoadjuvant treatment for the whole cohort of matched biopsy and resection specimens (*n* = 17) using Wilcoxon signed rank testing demonstrated a significant reduction in the DDIR score (Figure 2A; *P* < 0.001), an increase in mast cells (Figure 2B; *P* = 0.001) as well as consistent elevation of signatures indicative of the induction of angiogenesis and EMT (Figure 2C-E). A significant reduction in signatures representing genomic instability (Figure 2F; *P* < 0.001) and senescence (Figure 2G; *P* < 0.001) was also observed as well as an increase in transforming growth factor (TGF)-β signalling (Figure 2H; *P* < 0.001), which is proposed to drive immune evasion and the exclusion of T cells from the tumour microenvironment (TME).^31^^,^^32^ Finally, there was a reduction in two metabolic signatures following neoadjuvant therapy (Figure 2I-J; *P* < 0.001) with an increase in genes involved in myogenesis (Figure 2K; *P* < 0.001).

To further explore the associations between response/resistance to ICB, angiogenesis and EMT signature correlation analysis was carried out according to sample and treatment type (Figure 2L and M). For all pre-treatment biopsy samples, we observed a strong, positive correlation amongst signatures indicating response to ICB, which was less evident after treatment. In addition, there was increased correlation between resistance to ICB and EMT/angiogenesis signatures in both biopsy and resection specimens, indicating a potential common biology of therapeutic resistance.

Taken together, these results demonstrate that immune-high samples at biopsy show elevated acquired and innate immune signalling, immune checkpoint genes and signatures associated with response to ICB, which are markedly suppressed in matched post-treatment resection specimens. Combined with previous results which determined immune signatures as being predictive of PFS outcomes, we suggest that altered immune signalling after treatment potentially contributes to treatment resistance alongside EMT and angiogenesis signalling.

### HER2/EGFR inhibition induces an immunosuppressive microenvironment

**Figure 3:**
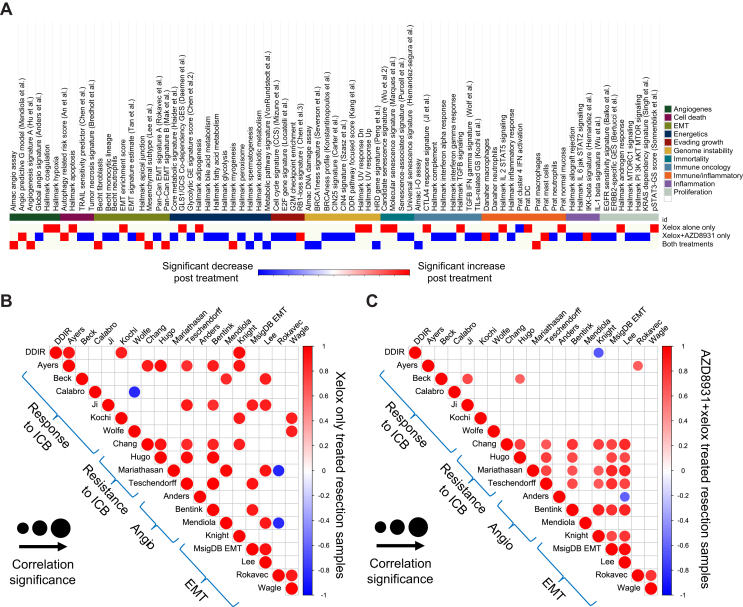
**Neoadjuvant therapy with Xelox + AZD8931 compared to Xelox alone alters EGFR/HER2 and immune signalling pathways.** (A) Heatmap highlighting gene signatures from their respective hallmarks which underwent a significant change from pre- to post-treatment using paired patient samples (*n* = 17) of which *n* = 11 received Xelox + AZD8931 and *n* = 6 received Xelox alone using Wilcoxon signed rank testing with false discovery rate (*P*-value <0.2). Unique signature changes are highlighted per treatment type as well as changes which were common across both treatment types, where red indicates a significant increase and blue indicates a significant decrease in signature scores from pre- to post-treatment. (B) Plots displaying significant results only from Spearman’s rank correlation testing (*P* < 0.05) of gene signatures relating to response/resistance to ICB, angiogenic or EMT signalling in resection samples treated with Xelox only (*n* = 6) and resection samples treated with Xelox + AZD8931 (*n* = 11), respectively. Increasing significance and strength of correlations are indicated by an increase in size and colour intensity of circles on the heatmap, where red indicates positive correlation and blue indicates negative correlation. EGFR, epidermal growth factor receptor; EMT, epithelial–mesenchymal transition; HER2, human epidermal growth factor receptor 2; ICB, immune checkpoint blockade.

To investigate changes in signalling pathways in each treatment arm following neoadjuvant therapy, alterations in signature scores for each cancer hallmark represented in the clara^T^ assay were compared. We observed 13 signature changes common to both treatment types reflecting increases in angiogenesis (2/13), EMT (3/13) and decreases in energetics (2/13), genome instability (3/13) and immune-oncology (1/13) (Figure 3A, Supplementary Table S9, available at https://doi.org/10.1016/j.esmoop.2024.103930). Considering the paired samples treated by Xelox alone (*n* = 6), 18 signatures were unique to this treatment arm, including increases in angiogenesis, cell death and EMT with the largest number of signatures belonging to the immuno-oncology/inflammation groups (Supplementary Table S10, available at https://doi.org/10.1016/j.esmoop.2024.103930). The paired samples which received a combination of Xelox + AZD8931 (*n* = 11) demonstrated 46 significant unique signature score changes with 39/46 signatures showing significant decreases in scores after treatment (Supplementary Table S11, available at https://doi.org/10.1016/j.esmoop.2024.103930). Most notably we observed 14 decreased scores derived from the immuno-oncology/inflammation hallmarks as well as a decrease in five ErbB downstream signalling pathways unique to Xelox + AZD8931, in keeping with the mechanism of action of this small-molecule pan-HER inhibitor.

Correlation analysis according to the treatment received indicated that the Xelox + AZD8931–treated resection specimens demonstrated more consistent correlation between resistance to ICB, angiogenesis and EMT gene expression signatures compared to patients treated with Xelox alone (Figure 3B and C).

Overall, Xelox + AZD8931 resulted in decreases in immuno-oncology/inflammation signature scores and ErbB downstream signalling suggesting selection of an immunosuppressive phenotype occurs following HER2/EGFR inhibition in combination with chemotherapy.

### Analysis of OAC biopsy samples reveals the importance of immune biology in defining response

To determine potential mechanisms of treatment resistance, we examined alterations in signature scores between pathological responders (*n* = 3) and non-responders (*n* = 22) and carried out gene-set enrichment analysis (GSEA) with a pre-ranked gene list derived from significance analysis of microarrays analysis.

**Figure 4:**
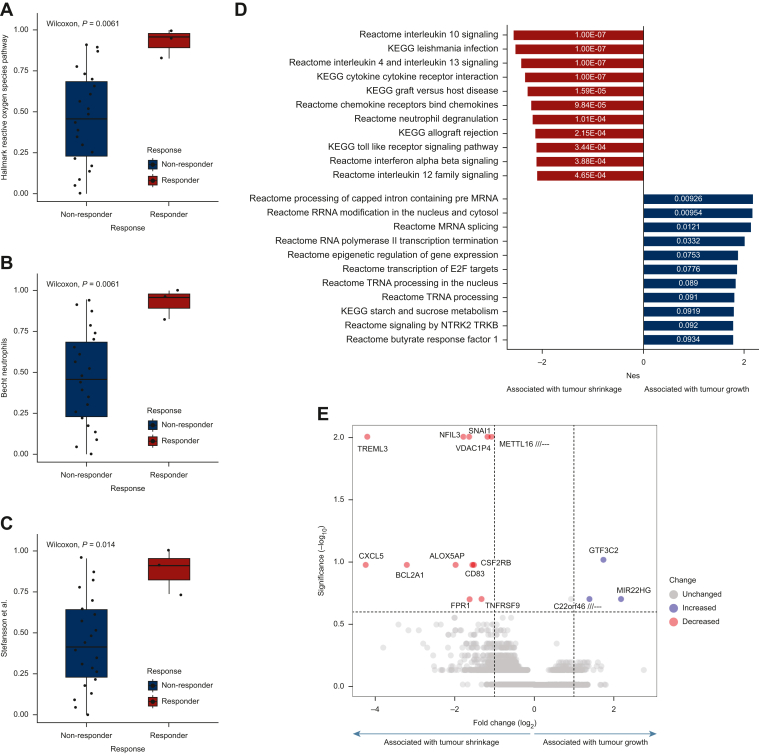
**Biological pathways mediating pathological responders to neoadjuvant therapy.** (A-C) Boxplots showing gene signature scores representing pathways significantly dysregulated between pathological responders (*n* = 3) and non-responders (*n* = 22) to neoadjuvant treatment at biopsy determined by Wilcoxon/Mann–Whitney *U* testing. (D) KEGG and Reactome pathways were significantly enriched, ranked by the normalised enrichment score and FDR *q*-value, when comparing pre-treatment biopsies of responders versus non-responders to neoadjuvant chemotherapy from GSEA, with pathways significantly associated with responders (*n* = 3) to neoadjuvant chemotherapy highlighted in red, and pathways significantly associated with non-responders (*n* = 22) to neoadjuvant chemotherapy highlighted in blue. (E) Volcano plot highlighting genes differentially expressed between responders (*n* = 3) and non-responders (*n* = 22) to neoadjuvant chemotherapy from significance analysis of microarrays analysis of respective patient biopsy samples (FDR *q*-value <0.2/−log10 *q*-value >0.6), where blue indicates genes significantly up-regulated in non-responders and red indicates genes significantly up-regulated in responders to neoadjuvant treatment. FDR, false discovery rate; GSEA, gene-set enrichment analysis.

Several molecular signalling pathways were found to be significantly altered in responders (Mandard score 1/2) when compared to non-responders (Mandard score 3-5) including up-regulated reactive oxygen species (*P* = 0.006), neutrophil infiltration (*P* = 0.006) and an angiogenesis signature which also contains genes reflecting EGFR/HER2 signalling (*P* = 0.014) (Figure 4A-C). GSEA was utilised to determine biological pathways which were enriched between responders and non-responders to neoadjuvant therapy (Figure 4D). Up-regulation of several inflammatory and interferon-related gene sets was associated with tumour downstaging, with the top 10 enriched pathways related to immune signalling (Supplementary Table S12, available at https://doi.org/10.1016/j.esmoop.2024.103930). Conversely, up-regulation of E2F targets, NTRK signalling and pathways involved in RNA processing were enriched in non-responders to neoadjuvant therapy (Supplementary Table S13, available at https://doi.org/10.1016/j.esmoop.2024.103930). In keeping with the GSEA results, the top ranked differentially expressed gene associated with tumour response was the inflammatory chemokine CXCL5, which has a key role in immune cell, particularly neutrophil, accumulation in tumours (Figure 4E and Supplementary Table S14, available at https://doi.org/10.1016/j.esmoop.2024.103930).^33^ Other up-regulated genes in pathological responders were associated with macrophage response (TREML3), leukotriene synthesis (ALOX5AP), NF-kappa B-mediated inflammatory response (BCL2A1) and an activator of transcription from the interleukin (IL)-3 promoter in T cells (NFIL3).

In summary, our results indicate that higher immune/inflammatory signalling at biopsy correlates with better response to treatment, whereas non-responders were enriched for cellular growth through RNA processing and the up-regulation of cyclins, cyclin-dependent kinases, checkpoint regulators and DNA repair proteins via the action of E2F transcription factors.

### Neoadjuvant therapy induces a reduction of tumour-infiltrating lymphocytes in the immune-high subgroup

To assess whether the reduction in immune signature and ICB gene expression following neoadjuvant therapy in the immune-high subgroup results in a reduction of TILs, we carried out digital pathology analysis of biopsy and resection specimens.

**Figure 5:**
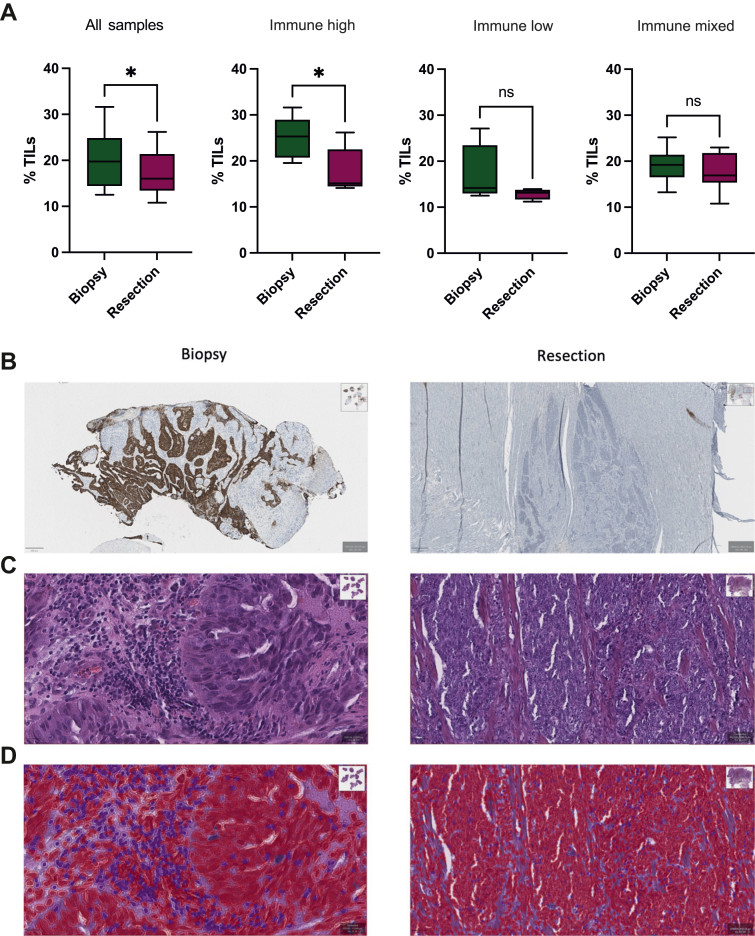
**Tumour-infiltrating lymphocytes and immune clusters.** (A) Alterations in TIL percentage following neoadjuvant therapy in the cohort as a whole and according to immune cluster designation (unpaired *t*-test; ∗ *P* < 0.05). Exemplar immune-high cluster case showing (B) positive HER2 expression [immunohistochemistry (IHC) 3+] in the biopsy specimen but loss of HER2 expression in the matched resection specimen. This was associated with a reduction in immune cell infiltration in the (C) unmodified and (D) enhanced haematoxylin and eosin–stained biopsy and resection specimens with TIL classification masks where TILs are identified using purple masks and all other cells, red. HER2, human epidermal growth factor receptor 2; TIL, tumour-infiltrating lymphocyte.

Taking the cohort as a whole, there was a significant reduction in TILs between pre-treatment biopsies and post-treatment resection specimens (*P* = 0.036) (Figure 5A). Subdividing the patients into the immune clusters defined previously showed that the immune-high tumours displayed a significant reduction in TILs (*P* = 0.03), whereas there was no similar reduction for immune-low or immune-mixed cases (*P* = 0.22 and *P* = 0.376, respectively). As an exemplar, one case (marked with ‘X’ on HER2 status on Figure 1) displayed IHC 3+ HER2 staining pre-treatment with Xelox + AZD8931, whereas the matched resection specimen did not express HER2 (Figure 5B). Pathological review of the biopsy and resection specimen illustrated a reduction in immune cell infiltration on their respective H&E-stained images (Figure 5C). Loss of TILs before and after treatment was quantified using image analysis to identify and count immune cell populations within the sample images (Figure 5D). Taken together these results suggest that the reduction in immune signature and ICB gene expression is matched by a reduction in TILs in response to neoadjuvant therapy in the immune-high subset of patients.

## Discussion

Gaining insight into the mechanisms of resistance to conventional and novel chemotherapeutics is crucial for improving outcomes in OAC. We have utilised pre-treatment endoscopic biopsies and resection specimens from the randomised phase II dose-expansion component of the DEBIOC trial to investigate transcriptional changes associated with treatment with oxaliplatin and capecitabine (Xelox) and the pan-HER inhibitor AZD8931. We have demonstrated that OAC can be stratified into three molecular subgroups based on the activation of innate immune signalling; immune-high, immune-low and immune-mixed. We have also shown that treatment with neoadjuvant chemotherapy induces common resistance mechanisms, such as angiogenesis and EMT signalling, as well as a reduction in DNA repair and cGAS-STING signalling. Following neoadjuvant therapy, a subset of samples with high immune signalling experienced a marked decline in the gene expression of signatures associated with immune signalling, ICB, angiogenesis and EMT in addition to immune checkpoints, such as PD-L1. This immune-high subgroup also contained both patients who were HER2 positive as well as two patients who attained a complete pathological response to neoadjuvant therapy. As expected, treatment with AZD8931 was associated with a reduction of expression of the EGFR, HER2 and AKT pathways but also displayed wider biological effects including either the direct inhibition of multiple pathways associated with immune and inflammatory signalling or selection for immunosuppressed subclones. GSEA showed that patients with a pathological response to treatment had increased immune signalling, whereas non-responders to neoadjuvant therapy were enriched for nucleotide repair and cellular growth through the action of E2F transcription factors. In summary, our results have shown that OAC can be subdivided into three immune-related clusters which undergo substantial change in the TME in response to neoadjuvant therapy. In particular, the pan-HER inhibitor AZD8931 may contribute to an immunosuppressive phenotype through inhibition of HER2/EGFR signalling.

Recent studies have revealed novel insights into the immuno-biology of OAC and the transcriptional changes which occur during neoadjuvant therapy. Naeini et al. described four OAC immune clusters using 115 pre-treatment biopsies derived from the phase II DOCTOR trial. The good prognosis ‘immune hot’ cluster showed increased expression of interferon and tumour necrosis factor-α as well as immune genes such as *CCL5*, *LAG3* and *IDO-1*, whereas the ‘immune-suppressed’ cluster was enriched with myeloid-derived cells, EMT signature expression and poor survival. Similarly, we have shown that the immune-high molecular subtype is characterised by up-regulation of immune signalling pathways, cytokines and immune checkpoint genes and the DDIR signature. Previous pre-clinical work in breast cancer has demonstrated that the underlying mechanism of innate immune response in DDIR-positive tumours is mediated by constitutive activation of the cGAS-STING pathway.^26^ In OAC, we previously showed a correlation between high DDIR score and pathological response, as well as infiltration with CD8+ lymphocytes and expression of PD-L1.^20^ Taken together, these factors indicate that tumours in the immune-high/DDIR-positive cluster display an inflamed but immune-restricted microenvironment, with increased immune cell infiltration co-existing with up-regulation of immune checkpoint genes. Parkes et al. utilised breast cancer samples before, during and after neoadjuvant chemotherapy and, in agreement with our study, demonstrated that mast cell and M2 signature scores were lower in the DDIR-positive subgroup alongside high macrophage scores.^34^ This pattern is in keeping with the known immunosuppressive and poor prognostic role of M2-polarised macrophages in OAC.^35^ In contrast to breast cancer, PD-L1 but not IDO-1 expression was high in the OAC immune-high/DDIR-positive cohort indicating potential differences in the immune microenvironment between the two tumour types. TGF-β signalling was also elevated in association with DDIR positivity, opposite to the observations in breast cancer where TGF-β was thought to contribute to immune exclusion in DDIR-negative tumours. However, the limited sample numbers in each study mean that any association should be interpreted with caution and will require validation in further, adequately powered cohorts.

In addition to differences in immune signalling at baseline between OAC and breast cancer, contrasting patterns were also observed following treatment with chemotherapy. Parkes et al. found that anthracycline-based regimens led to an induction of signatures predicting ICB response in DDIR-negative tumours as well as a marked increase in immune biologies, in particular interferon-α and -γ signalling.^34^ Importantly, no significant changes were seen in immune gene expression signalling before and after treatment in DDIR-positive tumours. In contrast, we observed a marked reduction of immune signalling, checkpoint genes and signatures following neoadjuvant therapy in the immune-high/DDIR-positive cluster. We also did not observe induction of immune signalling in the immune-low/mixed clusters following treatment, unlike the changes observed in breast cancer. These differing effects may in part be explained by the chemotherapies used in each study. Potent DNA-damaging agents, such as anthracyclines, as utilised in breast cancer, are capable of inducing the production of cytosolic DNA with subsequent activation of the cGAS-STING pathway in DNA repair-proficient models. *In vitro* screening and clinical trial data have also confirmed the ability of anthracyclines to induce immune checkpoint gene expression, potentially enhancing responses to ICB.^36^^,^^37^ In both of these breast cancer studies cisplatin, while not as potent as anthracyclines, was able to stimulate the development of an active immune microenvironment. Cisplatin directly modifies DNA through co-ordinate covalent bonds between DNA and the platinum moiety resulting in stalled/collapsed DNA replication forks, double strand breaks and activation of the innate immune signalling pathways.^38^ However, in the DEBIOC trial oxaliplatin was used as the chemotherapy backbone and, while it too causes DNA adducts, these are thought to be differentially recognised by a number of cellular proteins, including mismatch repair genes.^39^ Indeed, in colorectal cancer cases treated with oxaliplatin, the expected increased responses in DDIR-positive patients were not observed, with the addition of oxaliplatin to 5-fluorouracil more likely to benefit DDIR-negative patients.^40^ The use of oxaliplatin in the DEBIOC study may be insufficient to cause a high degree of DNA damage and so trigger the prolonged activation of innate immune signalling evident at the later time point of resection. It is also possible that the immune-high tumours may contain a higher proportion of chemosensitive clones and that following neoadjuvant therapy only residual, immune-quiescent cancer cells remain, leading to the observed collapse in immune signalling and reduction in TILs.

Translational analysis of sample sets from early-phase trials has a number of inherent strengths and weaknesses and our work is no exception. The strength of the work includes the use of pre- and post-treatment tissue samples, the ability to obtain high-quality gene expression data for all samples submitted for analysis and the first use of a novel pan-HER inhibitor in this disease setting. However, there were several limitations, with the foremost being the limited sample size, affecting the ability to draw definitive conclusions, and the use of non-standard neoadjuvant therapy. The lack of complementary sequencing or epigenetic data also limits our ability to accurately define putative resistance mechanisms in this cohort. Finally, the precision oncology landscape of gastro-oesophageal cancer has changed markedly during the lifetime of this project, and the addition of PD-L1 and Claudin 18.2 immunohistochemistry would help to further characterise the molecular subgroups identified but was not possible due to the limited amount of FFPE biopsy tissue available.^41^^,^^42^

Despite these potential drawbacks, our work provides novel insights—in particular, the identification of a subgroup of OAC cases characterised by HER2 positivity and high immune checkpoint expression. The results of the Keynote-811 study of the addition of the anti-HER2 monoclonal antibody trastuzumab and anti-PD-1 antibody pembrolizumab to platinum-based chemotherapy in OAC indicate the importance of targeting this subset of patients with a response in 73% of HER2-positive gastric and gastro-oesophageal junction adenocarcinomas and an improvement in OS (15.7-20 months) in patients with a PD-L1 combined positive score of ≥1.^43^ Crucially, several cases in our immune-high cohort were classified as HER2 negative but had strikingly similar gene expression profiles to the HER2-positive cases. This may reflect the known PD-L1-positive/HER2-negative subpopulation of OAC but could also indicate the limitations of HER2 IHC analysis of endoscopic biopsies to detect HER2-positive disease due to marked intra-tumoural heterogeneity. Recently, the analysis of 252 samples from the GO2 trial examining the role of dose-attenuated oxaliplatin-based chemotherapy in advanced gastro-oesophageal cancer has also shown improved radiological response and OS in DDIR-positive patients. EGFR amplification was associated with DDIR negativity and an immune-cold TME with the authors proposing that inhibition of EGFR could promote a pro-inflammatory state.^44^ This is in keeping with data from EGFR-mutated lung cancer where there is overproduction of negative immune modulators such as TGF-β and IL-10, directly suppressing NK, dendritic and cytotoxic T-cell function.^45^ EGFR inhibition led to an increase in the populations of CD8+ T cells, dendritic cells and M1-like tumour-associated macrophages in mouse lung cancer models.^46^ In addition, a subset of breast cancers with HER2 amplification exhibit an inflammatory phenotype but can also display impairment of the innate immune response through attenuation of STING activity and loss of phosphorylation of Tank-binding kinase 1.^47^ HER2 inhibition in these breast cancers can inhibit downstream PI3K-AKT signalling and induce antibody-dependent cell-mediated cytotoxicity, leading to up-regulation of programmed cell death protein 1 expression.^48^^,^^49^ This is in contrast to our observations where HER2 amplification is associated with an inflammatory phenotype characterised by activation of the cGAS-STING pathway and we observe that HER2/EGFR blockade may be immunosuppressive rather than increasing tumour immune cell populations. However, these results should be interpreted with caution due to the small sample size and combined inhibition of EGFR and HER2.

In conclusion, our translational analysis of the early-phase DEBIOC trial has identified three immune-related molecular subgroups with either elevated immune signalling associated with HER2 positivity and histopathological response, EGFR amplification with a more quiescent immune landscape or finally a HER2/EGFR-negative immunosuppressed phenotype. Neoadjuvant therapy results in an induction of common resistance signatures, such as angiogenesis and EMT, but also in the a marked reduction of immune/inflammatory signalling in the immune-high subgroup. OAC is a particularly heterogeneous disease with high degrees of intra-patient and intra-tumoural heterogeneity, so further analysis in larger datasets will be required to validate the clinically relevant themes which have emerged from our data. Overall, we have demonstrated the ability of neoadjuvant therapy to markedly modulate the immune microenvironment in OAC, which may inform the addition of novel immuno-therapeutics in this disease setting.
