# Supplementary material for: Immune microenvironment modulation following neoadjuvant therapy for oesophageal adenocarcinoma: a translational analysis of the DEBIOC clinical trial
Source: ESMO Open. 2024 Oct 11;9(11):103930. doi: 10.1016/j.esmoop.2024.103930 (PMC11693431; doi:10.1016/j.esmoop.2024.103930)
Supplement: Supplementary Tables and Figures [file mmc1.docx]

**Supplementary Tables and Figures**

**Supplementary Figure 1**. Trial Schema for the randomised phase II dose expansion study component of the DEBIOC trial with AZD8931 in combination with oxaliplatin and capecitabine (Xelox) chemotherapy in patients with resectable oesophageal or gastro-oesophageal junction (Siewert Type I and II) adenocarcinoma vs Xelox alone.

Oxaliplatin 130 mg/m^2^ D1 +

Capecitabine 1250 mg/m^2^ D1-21

2 cycles, n= 10

Oxaliplatin 130 mg/m^2^ D1 +

Capecitabine 1250 mg/m^2^ D1-21 +

AZD8931 20mg bd 4 days on/3 days off

2 cycles, n= 20

- Histologically confirmed adenocarcinoma of the oesophagus/gastro-oesophageal junction (Siewert Type I and II)
- cT2-4 /cN-any/cM0 or cT-any/cN+/cM0
- Deemed suitable for neo-adjuvant chemotherapy and medically/technically operable

Resection and Follow-up

Pre-treatment biopsy

Post-treatment resection

**DEBIOC Dose Expansion Cohort**

**n=30** Patients

n=20 Patients Xelox + AZD8931

n=10 Patients Xelox Alone

**OAC Biopsy and Resection Specimens**

**n=26** Patients (n=50 Samples)

Biopsy (n=27)*

Resection (n=23)

**Passed Pathology QC**

**n=25** Patients (n=43 Samples)

Biopsy (n=25)*

Resection (n=18)

**Paired Samples Post QC**

**n=17** Patients (n=34 Samples)

Biopsy (n=17)

Resection (n=17)

**Paired Samples Xelox + AZD8931**

**n=11** Patients (n=22 Samples)

Biopsy (n=11)

Resection (n=11)

**Paired Samples Xelox Alone**

**n=6** Patients (n=12 Samples)

Biopsy (n=6)

Resection (n=6)

**Excluded Patients n=4**

Samples not obtained at time of analysis

Did not proceed to surgery (n=3)

**Excluded Samples n=7**

Biopsy Failed Pathology QC (n=2)

Resection Failed Pathology QC (n=1)

No Resection Block Available (n=2)

Complete response (n=2)

**Unpaired Samples Post QC n=9**

Unpaired Biopsy Post QC

(n=8, 3 Xelox alone; 5 Xelox +AZD8931)*

Unpaired Resection Post QC

(n=1, Xelox +AZD8931)

*Duplicate biopsy present

**Supplementary Figure 2**. CONSORT diagram showing patient sample flow for the translational analysis of patients from the randomised phase II dose expansion study component for AZD8931 in combination with oxaliplatin and capecitabine (Xelox) chemotherapy in patients with resectable oesophageal of gastro-oesophageal junction (Siewert Type I and II) adenocarcinoma vs Xelox alone.


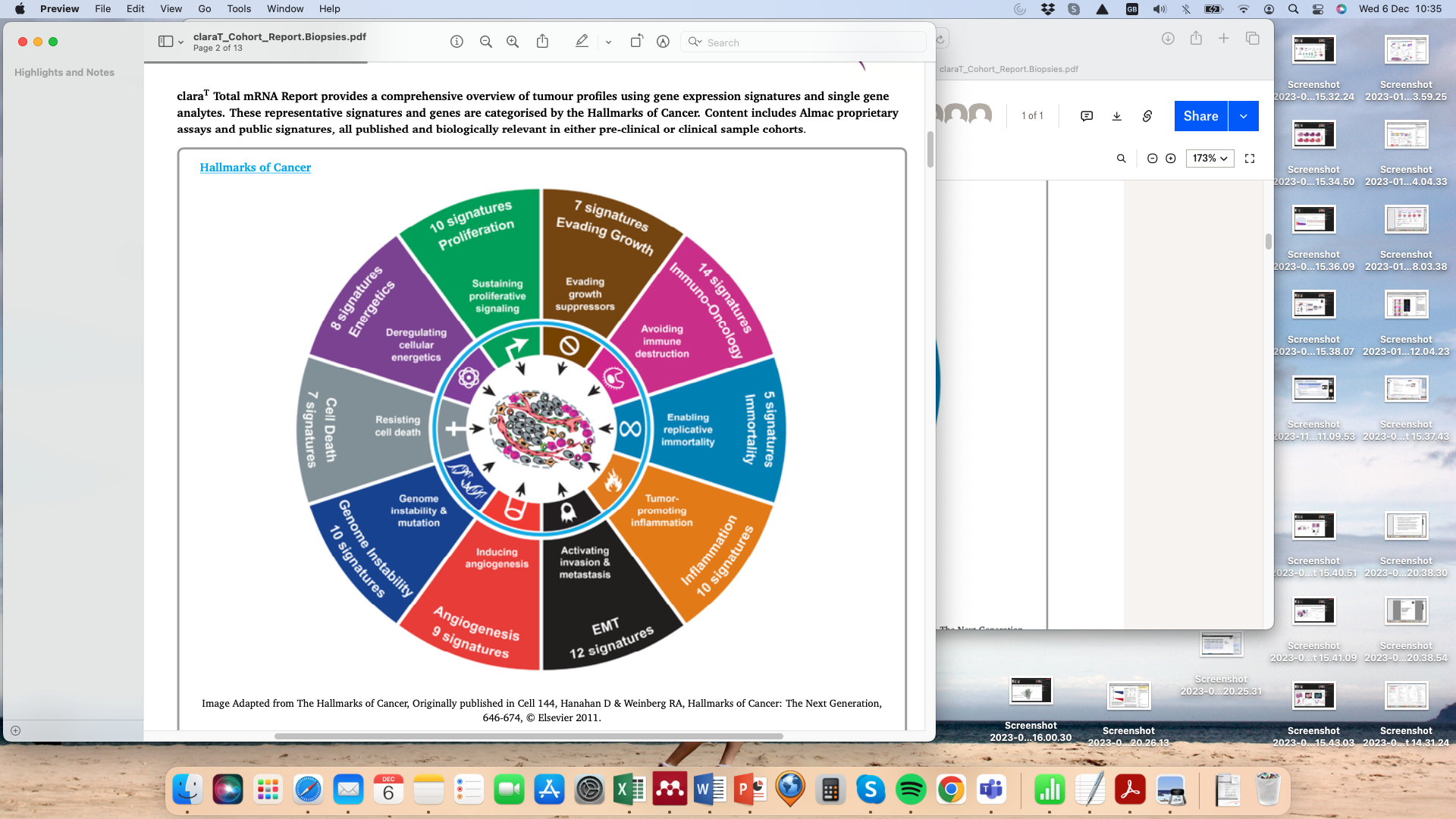


**Supplementary Figure 3.** The Almac Diagnostic Services claraT Total mRNA Report provides a comprehensive overview of tumour profiles using 92 gene expression signatures, 100 drug targets and 7337 single gene analytes. These representative signatures and genes are categorised by the Hallmarks of Cancer. Content includes Almac proprietary assays and public signatures, all published and biologically relevant in either pre-clinical or clinical sample. Adapted from The Hallmarks of Cancer, Originally published in Cell 144, Hanahan D & Weinberg RA, Hallmarks of Cancer: The Next Generation, 646-674, © Elsevier 2011

**Supplementary Figure 4.** Almac clara^T^ DDIR signature scores (percentile ranks) compared across clusters in the DEBIOC biopsy population (****P*< 0.001; Kruskal-Wallis test).

| **Supplementary Table 1** Clinicopathological characteristics of the DEBIOC study patients included in the translational analysis | | | | | | | | | | |
| --- | --- | --- | --- | --- | --- | --- | --- | --- | --- | --- |
|  |  | **All patients (n=25)** | | **Xelox (n=9)** | | | | **Xelox + AZD8931 (n=16)** | | **p value** |
|  |  | **n** | **%** | **n** | | **%** | | **n** | **%** | **(Fisher’s exact)** |
| **Sex** | | | | | | |  | |  |  |
|  | Male | 22 | 88 | 9 | | 100 | 13 | | 81 | 0.166 |
|  | Female | 3 | 12 | 0 | | 0 | 3 | | 19 |  |
| **Tumour Site** | | | | | | |  | |  |  |
|  | Oesophagus | 14 | 56 | 7 | | 78 | | 7 | 44 | 0.367 |
|  | GOJ, Siewert 1 | 2 | 8 | 1 | | 11 | | 1 | 6 |  |
|  | GOJ, Siewert 2 | 6 | 24 | 1 | | 11 | | 5 | 31 |  |
|  | Unknown | 3 | 12 | 0 | | 0 | | 3 | 19 |  |
| **T stage** | | | | | | | | | |  |
|  | T0/1 | 6 | 24 | 3 | | 33 | | 3 | 19 | 0.292 |
|  | T2 | 3 | 12 | 2 | | 22 | | 1 | 6 |  |
|  | T3 | 12 | 48 | 3 | | 33 | | 9 | 56 |  |
|  | T4 | 1 | 4 | 1 | | 11 | | 0 | 0 |  |
|  | No surgery | 3 | 12 | 0 | | 0 | | 3 | 19 |  |
| **N stage** | | | | | | |  | |  |  |
|  | N0 | 9 | 36 | 2 | | 22 | | 7 | 44 | 0.368 |
|  | N1 | 10 | 40 | 5 | | 56 | | 5 | 31 |  |
|  | N2 | 1 | 4 | 1 | | 11 | | 0 | 0 |  |
|  | N3 | 2 | 8 | 1 | | 11 | | 1 | 6 |  |
|  | No surgery | 3 | 12 | 0 | | 0 | | 3 | 19 |  |
| **Differentiation** | | | | | | |  | |  |  |
|  | Moderate | 12 | 48 | 4 | | 44 | | 8 | 5 | 0.678 |
|  | Poor | 7 | 28 | 3 | | 33 | | 4 | 25 |  |
|  | Unknown* | 6 | 24 | 2 | | 22 | | 4 | 25 |  |
| **Lymphovascular Invasion** | | | | | | |  | |  |  |
|  | Positive | 7 | 28 | 3 | | 33 | | 4 | 25 | 0.899 |
|  | Negative | 15 | 60 | 6 | | 67 | | 9 | 56 |  |
|  | No surgery | 3 | 12 | 0 | | 0 | | 3 | 19 |  |
| **Circumferential Resection Margin** | | | | | | |  | |  |  |
|  | Positive | 8 | 32 | 2 | | 22 | | 6 | 38 | 0.251 |
|  | Negative | 14 | 56 | 7 | | 78 | | 7 | 44 |  |
|  | No surgery | 3 | 12 | 0 | | 0 | | 3 | 19 |  |
| **Pathological Response** | | | | | | |  | |  |  |
|  | Responder | 3 | 12 | 1 | | 11 | | 2 | 13 | 0.948 |
|  | Non-Responder | 17 | 68 | 6 | | 67 | | 11 | 69 |  |
|  | No surgery | 5 | 20 | 2 | | 22 | | 3 | 18 |  |
| **HER2 Classification (Biopsy n=24)** | | | | | | |  | |  |  |
|  | 0 | 13 | 54 | 5 | | 56 | | 8 | 53 | 0.787 |
|  | 1+ | 5 | 21 | 1 | | 11 | | 4 | 27 |  |
|  | 2+ | 4 | 17 | 2 | | 22 | | 2 | 13 |  |
|  | 3+ | 2 | 8 | 1 | | 11 | | 1 | 7 |  |
| **HER2 Classification (Resection n=18)** | | | | | | |  | |  |  |
|  | 0 | 8 | 44 | 3 | | 50 | | 5 | 42 | 0.371 |
|  | 1+ | 7 | 39 | 2 | | 33 | | 5 | 42 |  |
|  | 2+ | 1 | 6 | 1 | | 17 | | 0 | 0 |  |
|  | 3+ | 2 | 11 | 0 | | 0 | | 2 | 17 |  |
| **EGFR FISH Classification (Biopsy n=24)** | | | | | | |  | |  |  |
|  | Positive | 8 | 33 | 3 | | 33 | | 5 | 33 | 0.99 |
|  | Negative | 11 | 46 | 4 | | 44 | | 7 | 47 |  |
|  | Fail | 5 | 21 | 2 | | 22 | | 3 | 20 |  |
|  | *3 patients did not proceed to surgery and 3 patients obtained TRG 1/2 | | | |  | |  | |  |  |

**Supplementary Table 2.** EGFR FISH results and status according to cluster in the DEBIOC population.

|  | **I**mmune High  (n= 4) | Immune Low (n=7) |  | Immune Mixed (n=9) |  | p-value  (Fisher’s exact) |
| --- | --- | --- | --- | --- | --- | --- |
| **EGFR FISH Result** |  |  |  |  |  |  |
| Amplified (FISH positive) | 0 | 0 |  | 2 (22%) |  | 0.288 |
| High polysomy (FISH positive) | 1 (25%) | 1 (14%) |  | 5 (56%) |  |  |
| High trisomy (FISH negative) | 0 | 0 |  | 0 |  |  |
| Low polysomy (FISH negative) | 1 (25%) | 3 (43%) |  | 1 (11%) |  |  |
| Low trisomy (FISH negative) | 2 (50%) | 2 (29%) |  | 1 (11%) |  |  |
| Disomy (FISH negative) | 0 | 1 (14%) |  | 0 |  |  |
| **EGFR FISH Status** |  |  |  |  |  |  |
| FISH positive | 1 (25%) | 1 (14%) |  | 7 (78%) |  | 0.027 |
| FISH negative | 3 (75%) | 6 (86%) |  | 2 (22%) |  |  |

| **Supplementary Table 3.** Comparison of gene signatures across the three immune clusters in the biopsy samples | | | | | | | | | | | | | | | | | | | |  | |
| --- | --- | --- | --- | --- | --- | --- | --- | --- | --- | --- | --- | --- | --- | --- | --- | --- | --- | --- | --- | --- | --- |
|  |  | Cluster 1 V Other Clusters Mann-Whitney U Testing | | | | | | Cluster 2 V Other Clusters Mann-Whitney U Testing | | | | | Cluster 3 V Other Clusters Mann-Whitney U Testing | | | | |  | |  | |
| Category | Signatures | Median Cluster1 | Median Other | Higher In | p-value | FDR | Median Cluster2 | | Median Other | Higher In | p-value | FDR | Median Cluster3 | Median Other | Higher In | p-value | FDR | Kruskal-Wallis | |  |  |
| Acquired Immune | CD45 | 0.935 | 0.391 | Cluster1 | 0.001 | 0.005 | 0.478 | | 0.674 | Other | 0.193 | 0.388 | 0.326 | 0.609 | Other | 0.097 | 0.217 | 0.003 | | ** |  |
| Acquired Immune | B | 0.261 | 0.522 | Other | 0.221 | 0.328 | 0.217 | | 0.630 | Other | 0.004 | 0.027 | 0.761 | 0.217 | Cluster3 | 0.000 | 0.005 | 0.001 | | ** |  |
| Acquired Immune | T | 0.500 | 0.478 | Cluster1 | 0.475 | 0.529 | 0.130 | | 0.587 | Other | 0.002 | 0.027 | 0.609 | 0.333 | Cluster3 | 0.052 | 0.128 | 0.014 | | * |  |
| Acquired Immune | CD8 | 0.848 | 0.391 | Cluster1 | 0.003 | 0.012 | 0.174 | | 0.630 | Other | 0.005 | 0.030 | 0.500 | 0.609 | Other | 0.979 | 0.979 | 0.004 | | ** |  |
| Acquired Immune | Cytotoxic | 0.935 | 0.435 | Cluster1 | 0.008 | 0.025 | 0.348 | | 0.587 | Other | 0.289 | 0.445 | 0.500 | 0.739 | Other | 0.201 | 0.318 | 0.028 | | * |  |
| Acquired Immune | Exhausted | 0.891 | 0.435 | Cluster1 | 0.007 | 0.023 | 0.217 | | 0.630 | Other | 0.029 | 0.102 | 0.543 | 0.478 | Cluster3 | 0.810 | 0.845 | 0.013 | | * |  |
| Acquired Immune | Th1 | 0.696 | 0.435 | Cluster1 | 0.221 | 0.328 | 0.217 | | 0.630 | Other | 0.012 | 0.057 | 0.630 | 0.348 | Cluster3 | 0.270 | 0.401 | 0.042 | | * |  |
| Acquired Immune | Treg | 0.609 | 0.435 | Cluster1 | 0.828 | 0.864 | 0.217 | | 0.587 | Other | 0.055 | 0.142 | 0.543 | 0.435 | Cluster3 | 0.137 | 0.268 | 0.138 | |  |  |
| Innate Immune | DC | 0.804 | 0.435 | Cluster1 | 0.138 | 0.233 | 0.435 | | 0.522 | Other | 0.701 | 0.780 | 0.435 | 0.522 | Other | 0.376 | 0.512 | 0.309 | |  |  |
| Innate Immune | Macrophages | 0.913 | 0.391 | Cluster1 | 0.001 | 0.006 | 0.348 | | 0.674 | Other | 0.096 | 0.235 | 0.543 | 0.478 | Cluster3 | 0.221 | 0.338 | 0.004 | | ** |  |
| Innate Immune | Neutrophils | 0.582 | 0.391 | Cluster1 | 0.121 | 0.212 | 0.391 | | 0.539 | Other | 0.574 | 0.669 | 0.413 | 0.556 | Other | 0.437 | 0.579 | 0.282 | |  |  |
| Innate Immune | NK.CD56dim | 0.609 | 0.522 | Cluster1 | 0.437 | 0.498 | 0.130 | | 0.674 | Other | 0 | 0.000 | 0.674 | 0.261 | Cluster3 | 0.007 | 0.039 | 0.001 | | ** |  |
| Innate Immune | NK | 0.457 | 0.565 | Other | 0.366 | 0.457 | 0.130 | | 0.587 | Other | 0.004 | 0.027 | 0.761 | 0.304 | Cluster3 | 0.000 | 0.011 | 0.002 | | ** |  |
| Innate Immune | Mast | 0.220 | 0.609 | Other | 0.092 | 0.188 | 0.261 | | 0.500 | Other | 0.389 | 0.502 | 0.652 | 0.222 | Cluster3 | 0.022 | 0.072 | 0.062 | |  |  |
| Innate Immune | M2 | 0.316 | 0.478 | Other | 0.400 | 0.479 | 0.565 | | 0.457 | Cluster2 | 0.244 | 0.428 | 0.457 | 0.522 | Other | 0.769 | 0.845 | 0.425 | |  |  |
| Interferon Signalling | Complement | 0.935 | 0.391 | Cluster1 | 0.001 | 0.004 | 0.435 | | 0.630 | Other | 0.318 | 0.445 | 0.348 | 0.783 | Other | 0.041 | 0.107 | 0.002 | | ** |  |
| Interferon Signalling | IL2.STAT5 | 0.870 | 0.391 | Cluster1 | 0.000 | 0.004 | 0.391 | | 0.630 | Other | 0.110 | 0.257 | 0.391 | 0.667 | Other | 0.186 | 0.304 | 0.004 | | ** |  |
| Interferon Signalling | Inflammatory Response | 0.907 | 0.391 | Cluster1 | 0.001 | 0.006 | 0.348 | | 0.543 | Other | 0.198 | 0.388 | 0.413 | 0.652 | Other | 0.168 | 0.295 | 0.009 | | ** |  |
| Interferon Signalling | IFNA | 0.935 | 0.391 | Cluster1 | 0.000 | 0.004 | 0.435 | | 0.609 | Other | 0.289 | 0.445 | 0.348 | 0.739 | Other | 0.041 | 0.107 | 0.002 | | ** |  |
| Interferon Signalling | IFNG | 0.913 | 0.391 | Cluster1 | 0.003 | 0.012 | 0.435 | | 0.630 | Other | 0.318 | 0.445 | 0.370 | 0.565 | Other | 0.109 | 0.231 | 0.011 | | * |  |
| Interferon Signalling | TGFB | 0.761 | 0.391 | Cluster1 | 0.011 | 0.031 | 0.435 | | 0.511 | Other | 0.976 | 0.997 | 0.326 | 0.652 | Other | 0.030 | 0.091 | 0.028 | | * |  |
| Immune Checkpoints | PDL1 | 0.913 | 0.391 | Cluster1 | 0.002 | 0.009 | 0.478 | | 0.565 | Other | 0.739 | 0.794 | 0.304 | 0.696 | Other | 0.021 | 0.072 | 0.006 | | ** |  |
| Immune Checkpoints | CD40 | 0.804 | 0.391 | Cluster1 | 0.011 | 0.031 | 0.304 | | 0.674 | Other | 0.055 | 0.142 | 0.457 | 0.565 | Other | 0.728 | 0.830 | 0.024 | | * |  |
| Immune Checkpoints | CD27 | 0.461 | 0.522 | Other | 0.926 | 0.926 | 0.174 | | 0.630 | Other | 0.001 | 0.017 | 0.717 | 0.304 | Cluster3 | 0.007 | 0.039 | 0.005 | | ** |  |
| Immune Checkpoints | CTLA4 | 0.565 | 0.435 | Cluster1 | 0.828 | 0.864 | 0.565 | | 0.457 | Cluster2 | 0.574 | 0.669 | 0.391 | 0.565 | Other | 0.470 | 0.606 | 0.735 | |  |  |
| Immune Checkpoints | LAG3 | 0.696 | 0.435 | Cluster1 | 0.121 | 0.212 | 0.217 | | 0.587 | Other | 0.041 | 0.132 | 0.543 | 0.522 | Cluster3 | 0.650 | 0.758 | 0.078 | |  |  |
| Immune Checkpoints | IDO1 | 0.283 | 0.609 | Other | 0.080 | 0.170 | 0.304 | | 0.543 | Other | 0.158 | 0.338 | 0.761 | 0.304 | Cluster3 | 0.004 | 0.036 | 0.017 | | * |  |
| Immune Checkpoints | HAVCR2 | 0.650 | 0.478 | Cluster1 | 0.303 | 0.401 | 0.391 | | 0.543 | Other | 1.000 | 1.000 | 0.522 | 0.522 | Cluster3 | 0.376 | 0.512 | 0.513 | |  |  |
| Immune Checkpoints | PD1 | 0.413 | 0.522 | Other | 0.926 | 0.926 | 0.217 | | 0.500 | Other | 0.270 | 0.445 | 0.543 | 0.348 | Cluster3 | 0.376 | 0.512 | 0.494 | |  |  |
| ICB Response | DDIR | 0.935 | 0.391 | Cluster1 | 0.001 | 0.004 | 0.174 | | 0.674 | Other | 0.003 | 0.027 | 0.543 | 0.478 | Cluster3 | 0.807 | 0.845 | 0.000 | | *** |  |
| ICB Response | Ayers | 0.929 | 0.391 | Cluster1 | 0.0000113 | 0.001 | 0.261 | | 0.609 | Other | 0.055 | 0.142 | 0.457 | 0.739 | Other | 0.186 | 0.304 | 0.001 | | ** |  |
| ICB Response | Beck | 0.891 | 0.391 | Cluster1 | 0.000 | 0.010 | 0.174 | | 0.587 | Other | 0.014 | 0.058 | 0.478 | 0.478 | Other | 0.810 | 0.845 | 0.005 | | ** |  |
| ICB Response | Calabro | 0.717 | 0.435 | Cluster1 | 0.198 | 0.313 | 0.130 | | 0.674 | Other | 0.000 | 0.000 | 0.630 | 0.261 | Cluster3 | 0.022 | 0.072 | 0.001 | | ** |  |
| ICB Response | Ji | 0.935 | 0.391 | Cluster1 | 0.001 | 0.005 | 0.261 | | 0.674 | Other | 0.014 | 0.058 | 0.543 | 0.478 | Cluster3 | 0.532 | 0.659 | 0.001 | | ** |  |
| ICB Response | Kochi | 0.935 | 0.391 | Cluster1 | 0.000 | 0.004 | 0.435 | | 0.587 | Other | 0.318 | 0.445 | 0.348 | 0.696 | Other | 0.032 | 0.091 | 0.001 | | ** |  |
| ICB Response | Wolfe | 0.783 | 0.435 | Cluster1 | 0.059 | 0.138 | 0.304 | | 0.630 | Other | 0.220 | 0.400 | 0.522 | 0.522 | Cluster3 | 0.650 | 0.758 | 0.132 | |  |  |
| ICB Resistance | Chang | 0.587 | 0.478 | Cluster1 | 0.733 | 0.798 | 0.783 | | 0.348 | Cluster2 | 0.012 | 0.057 | 0.283 | 0.739 | Other | 0.010 | 0.043 | 0.019 | | * |  |
| ICB Resistance | Hugo | 0.826 | 0.391 | Cluster1 | 0.017 | 0.044 | 0.522 | | 0.413 | Cluster2 | 0.458 | 0.576 | 0.261 | 0.696 | Other | 0.005 | 0.039 | 0.014 | | * |  |
| ICB Resistance | Mariathasan | 0.652 | 0.435 | Cluster1 | 0.303 | 0.401 | 0.739 | | 0.370 | Cluster2 | 0.029 | 0.102 | 0.304 | 0.739 | Other | 0.003 | 0.036 | 0.013 | | * |  |
| ICB Resistance | Teschendorff | 0.674 | 0.435 | Cluster1 | 0.106 | 0.208 | 0.783 | | 0.390 | Cluster2 | 0.047 | 0.142 | 0.283 | 0.739 | Other | 0.001 | 0.012 | 0.006 | | ** |  |
| Angiogenesis | Gourley | 0.348 | 0.478 | Other | 0.373 | 0.457 | 0.652 | | 0.413 | Cluster2 | 0.318 | 0.445 | 0.413 | 0.565 | Other | 0.913 | 0.932 | 0.492 | |  |  |
| Angiogenesis | Anders | 0.609 | 0.391 | Cluster1 | 0.437 | 0.498 | 0.609 | | 0.457 | Cluster2 | 0.389 | 0.502 | 0.326 | 0.609 | Other | 0.137 | 0.268 | 0.314 | |  |  |
| Angiogenesis | Bentink | 0.653 | 0.435 | Cluster1 | 0.121 | 0.212 | 0.652 | | 0.435 | Cluster2 | 0.220 | 0.400 | 0.304 | 0.652 | Other | 0.011 | 0.047 | 0.042 | | * |  |
| Angiogenesis | Mendiola | 0.254 | 0.522 | Other | 0.274 | 0.383 | 0.435 | | 0.522 | Other | 0.657 | 0.749 | 0.652 | 0.391 | Cluster3 | 0.168 | 0.295 | 0.332 | |  |  |
| EMT | Knight | 0.804 | 0.478 | Cluster1 | 0.274 | 0.383 | 0.522 | | 0.522 | Other | 0.745 | 0.794 | 0.457 | 0.609 | Other | 0.538 | 0.659 | 0.519 | |  |  |
| EMT | MsigDB.EMT | 0.609 | 0.435 | Cluster1 | 0.176 | 0.288 | 0.696 | | 0.457 | Cluster2 | 0.125 | 0.278 | 0.326 | 0.652 | Other | 0.008 | 0.039 | 0.033 | | * |  |
| EMT | Lee | 0.717 | 0.391 | Cluster1 | 0.069 | 0.154 | 0.522 | | 0.500 | Cluster2 | 0.929 | 0.969 | 0.283 | 0.609 | Other | 0.098 | 0.217 | 0.130 | |  |  |
| EMT | Rokavec | 0.761 | 0.391 | Cluster1 | 0.036 | 0.088 | 0.652 | | 0.478 | Cluster2 | 0.389 | 0.502 | 0.326 | 0.696 | Other | 0.008 | 0.039 | 0.024 | | * |  |
| EMT | Wagle | 0.609 | 0.391 | Cluster1 | 0.366 | 0.457 | 0.609 | | 0.457 | Cluster2 | 0.495 | 0.607 | 0.348 | 0.609 | Other | 0.152 | 0.286 | 0.334 | |  |  |
| Significance: * Kruskal-Wallis p <0.05; ** Kruskal-Wallis p <0.01; ***Kruskal-Wallis p <0.001. | | | | | | | | | | | | | | | | | | |  |  |  |

| **Signature** | **Reported Biology** | **Hallmark** | **AUC** |
| --- | --- | --- | --- |
| Chang_et_al | TCGA Fibroblast CSR | Inflammation | 0.833 |
| Yuan_et_al | M1/M2 gene expression signature | Inflammation | 0.792 |
| Knijenburg_et_al | TP53 Classifier | Cell Death/Evading Growth | 0.75 |
| Prat_T_helper_cells | T Helper Cells | Immuno-Oncology/Inflammation | 0.75 |
| Glinsky_et_al | MTTS Signature | Angiogenesis | 0.692 |
| Prat_Normal_mucosa | Normal Mucosa | Immuno-Oncology/Inflammation | 0.692 |
| Chen_et_al2 | Glycolytic GE Signature Score | Energetics | 0.658 |
| Locatelli_et_al | E2F Gen Signature | Evading Growth | 0.658 |
| Teschendorff_et_al | TCGA TGF Beta Signature | Immuno-oncology/Inflammation | 0.658 |
| Jung_et_al | MYC Activity Signature | Proliferation | 0.658 |
| Rokavec_et_al | Pan-Can EMT Signature A | EMT | 0.65 |

**Supplementary Table 4**: Area under the Receiver Operating Curve (AUC-ROC) Scores for Signature Scores at Biopsy. Listed clara^T^ signatures which had a score of > 0.65 from ROC-AUC analysis based on whether relapse had occurred or not at 24 months.

| **Supplementary Table 5.** Comparison of gene signatures across the three immune clusters in the resection samples | |
| --- | --- |
| \|  \| \| Cluster 1 V Other Clusters Mann-Whitney U Testing \| \| \| \| \| Cluster 2 V Other Clusters Mann-Whitney U Testing \| \| \| \| \| Cluster 3 V Other Clusters Mann-Whitney U Testing \| \| \| \| \|  \|  \| \| --- \| --- \| --- \| --- \| --- \| --- \| --- \| --- \| --- \| --- \| --- \| --- \| --- \| --- \| --- \| --- \| --- \| --- \| --- \| \| Category \| Signatures \| Median Cluster1 \| Median Other \| Higher In \| p-value \| FDR \| Median Cluster2 \| Median Other \| Higher In \| p-value \| FDR \| Median Cluster3 \| Median Other \| Higher In \| p-value \| FDR \| Kruskal-Wallis p-value \|  \| \| Acquired Immune \| CD45 \| 0.167 \| 0.528 \| Other \| 0.068 \| 0.207 \| 0.639 \| 0.333 \| Cluster2 \| 0.163 \| 0.666 \| 0.472 \| 0.444 \| Cluster3 \| 0.887 \| 0.962 \| 0.100 \|  \| \| Acquired Immune \| B \| 0.722 \| 0.472 \| Cluster1 \| 0.591 \| 0.828 \| 0.528 \| 0.500 \| Cluster2 \| 0.703 \| 0.976 \| 0.444 \| 0.611 \| Other \| 0.962 \| 0.962 \| 0.784 \|  \| \| Acquired Immune \| T \| 0.444 \| 0.444 \| Other \| 0.768 \| 0.896 \| 0.556 \| 0.389 \| Cluster2 \| 0.703 \| 0.976 \| 0.333 \| 0.500 \| Other \| 0.536 \| 0.962 \| 0.792 \|  \| \| Acquired Immune \| CD8 \| 0.833 \| 0.417 \| Cluster1 \| 0.244 \| 0.460 \| 0.472 \| 0.444 \| Cluster2 \| 0.956 \| 0.976 \| 0.417 \| 0.722 \| Other \| 0.417 \| 0.962 \| 0.442 \|  \| \| Acquired Immune \| Cytotoxic \| 0.333 \| 0.472 \| Other \| 0.676 \| 0.850 \| 0.472 \| 0.444 \| Cluster2 \| 0.871 \| 0.976 \| 0.472 \| 0.389 \| Cluster3 \| 0.887 \| 0.962 \| 0.876 \|  \| \| Acquired Immune \| Exhausted \| 0.444 \| 0.444 \| Other \| 0.859 \| 0.956 \| 0.222 \| 0.500 \| Other \| 0.412 \| 0.841 \| 0.528 \| 0.278 \| Cluster3 \| 0.601 \| 0.962 \| 0.663 \|  \| \| Acquired Immune \| Th1 \| 0.722 \| 0.528 \| Cluster1 \| 0.300 \| 0.525 \| 0.306 \| 0.611 \| Other \| 0.245 \| 0.707 \| 0.583 \| 0.444 \| Cluster3 \| 0.887 \| 0.962 \| 0.323 \|  \| \| Acquired Immune \| Treg \| 0.611 \| 0.472 \| Cluster1 \| 1.000 \| 1.000 \| 0.528 \| 0.444 \| Cluster2 \| 0.785 \| 0.976 \| 0.417 \| 0.556 \| Other \| 0.813 \| 0.962 \| 0.940 \|  \| \| Innate Immune \| DC \| 0.111 \| 0.528 \| Other \| 0.197 \| 0.386 \| 0.500 \| 0.500 \| Other \| 0.785 \| 0.976 \| 0.528 \| 0.389 \| Cluster3 \| 0.475 \| 0.962 \| 0.383 \|  \| \| Innate Immune \| Macrophages \| 0.500 \| 0.583 \| Other \| 0.676 \| 0.850 \| 0.861 \| 0.500 \| Cluster2 \| 0.130 \| 0.638 \| 0.500 \| 0.667 \| Other \| 0.364 \| 0.962 \| 0.283 \|  \| \| Innate Immune \| Neutrophils \| 0.444 \| 0.528 \| Other \| 0.953 \| 0.973 \| 0.361 \| 0.500 \| Other \| 0.549 \| 0.927 \| 0.528 \| 0.444 \| Cluster3 \| 0.536 \| 0.962 \| 0.756 \|  \| \| Innate Immune \| NK.CD56dim \| 0.833 \| 0.417 \| Cluster1 \| 0.068 \| 0.207 \| 0.167 \| 0.667 \| Other \| 0.045 \| 0.638 \| 0.528 \| 0.556 \| Other \| 0.813 \| 0.962 \| 0.046 \| * \| \| Innate Immune \| NK \| 0.944 \| 0.417 \| Cluster1 \| 0.047 \| 0.192 \| 0.139 \| 0.667 \| Other \| 0.010 \| 0.494 \| 0.611 \| 0.278 \| Cluster3 \| 0.601 \| 0.962 \| 0.016 \| * \| \| Innate Immune \| Mast \| 0.056 \| 0.556 \| Other \| 0.003 \| 0.072 \| 0.806 \| 0.389 \| Cluster2 \| 0.130 \| 0.638 \| 0.472 \| 0.222 \| Cluster3 \| 0.536 \| 0.962 \| 0.019 \| * \| \| Innate Immune \| M2 \| 0.333 \| 0.528 \| Other \| 0.859 \| 0.956 \| 0.583 \| 0.500 \| Cluster2 \| 0.703 \| 0.976 \| 0.528 \| 0.389 \| Cluster3 \| 0.887 \| 0.962 \| 0.894 \|  \| \| Interferon Signalling \| Complement \| 0.111 \| 0.583 \| Other \| 0.021 \| 0.176 \| 0.417 \| 0.556 \| Other \| 1.000 \| 1.000 \| 0.639 \| 0.222 \| Cluster3 \| 0.088 \| 0.962 \| 0.064 \|  \| \| Interferon Signalling \| IL2.STAT5 \| 0.111 \| 0.528 \| Other \| 0.032 \| 0.176 \| 0.583 \| 0.333 \| Cluster2 \| 0.163 \| 0.666 \| 0.444 \| 0.444 \| Cluster3 \| 0.740 \| 0.962 \| 0.064 \|  \| \| Interferon Signalling \| Inflammatory Response \| 0.111 \| 0.500 \| Other \| 0.121 \| 0.269 \| 0.500 \| 0.389 \| Cluster2 \| 0.956 \| 0.976 \| 0.528 \| 0.444 \| Cluster3 \| 0.270 \| 0.962 \| 0.249 \|  \| \| Interferon Signalling \| IFNA \| 0.500 \| 0.417 \| Cluster1 \| 0.768 \| 0.896 \| 0.500 \| 0.444 \| Cluster2 \| 0.871 \| 0.976 \| 0.417 \| 0.500 \| Other \| 0.962 \| 0.962 \| 0.923 \|  \| \| Interferon Signalling \| IFNG \| 0.222 \| 0.528 \| Other \| 0.432 \| 0.706 \| 0.556 \| 0.500 \| Cluster2 \| 0.624 \| 0.976 \| 0.528 \| 0.389 \| Cluster3 \| 0.887 \| 0.962 \| 0.637 \|  \| \| Interferon Signalling \| TGFB \| 0.222 \| 0.583 \| Other \| 0.156 \| 0.332 \| 0.722 \| 0.389 \| Cluster2 \| 0.130 \| 0.638 \| 0.500 \| 0.444 \| Cluster3 \| 0.887 \| 0.962 \| 0.148 \|  \| \| Immune Checkpoints \| PDL1 \| 0.444 \| 0.444 \| Other \| 0.768 \| 0.896 \| 0.306 \| 0.500 \| Other \| 0.477 \| 0.866 \| 0.528 \| 0.389 \| Cluster3 \| 0.364 \| 0.962 \| 0.610 \|  \| \| Immune Checkpoints \| CD40 \| 0.167 \| 0.583 \| Other \| 0.032 \| 0.176 \| 0.750 \| 0.389 \| Cluster2 \| 0.079 \| 0.638 \| 0.500 \| 0.500 \| Other \| 0.962 \| 0.962 \| 0.043 \| * \| \| Immune Checkpoints \| CD27 \| 0.389 \| 0.583 \| Other \| 0.509 \| 0.804 \| 0.417 \| 0.556 \| Other \| 0.871 \| 0.976 \| 0.583 \| 0.389 \| Cluster3 \| 0.475 \| 0.962 \| 0.684 \|  \| \| Immune Checkpoints \| CTLA4 \| 0.444 \| 0.583 \| Other \| 0.953 \| 0.973 \| 0.639 \| 0.500 \| Cluster2 \| 0.871 \| 0.976 \| 0.556 \| 0.556 \| Other \| 0.962 \| 0.962 \| 0.972 \|  \| \| Immune Checkpoints \| LAG3 \| 0.889 \| 0.444 \| Cluster1 \| 0.091 \| 0.235 \| 0.583 \| 0.444 \| Cluster2 \| 0.956 \| 0.976 \| 0.361 \| 0.667 \| Other \| 0.230 \| 0.962 \| 0.198 \|  \| \| Immune Checkpoints \| IDO1 \| 0.944 \| 0.472 \| Cluster1 \| 0.047 \| 0.192 \| 0.250 \| 0.667 \| Other \| 0.045 \| 0.638 \| 0.611 \| 0.500 \| Cluster3 \| 0.887 \| 0.962 \| 0.038 \| * \| \| Immune Checkpoints \| HAVCR2 \| 0.111 \| 0.528 \| Other \| 0.021 \| 0.176 \| 0.528 \| 0.444 \| Cluster2 \| 0.703 \| 0.976 \| 0.528 \| 0.222 \| Cluster3 \| 0.193 \| 0.962 \| 0.076 \|  \| \| Immune Checkpoints \| PD1 \| 0.778 \| 0.472 \| Cluster1 \| 0.068 \| 0.207 \| 0.389 \| 0.611 \| Other \| 0.296 \| 0.763 \| 0.472 \| 0.667 \| Other \| 0.669 \| 0.962 \| 0.134 \|  \| \| ICB Response \| DDIR \| 0.667 \| 0.417 \| Cluster1 \| 0.197 \| 0.386 \| 0.250 \| 0.556 \| Other \| 0.350 \| 0.841 \| 0.500 \| 0.500 \| Other \| 0.887 \| 0.962 \| 0.302 \|  \| \| ICB Response \| Ayers \| 0.611 \| 0.472 \| Cluster1 \| 0.591 \| 0.828 \| 0.389 \| 0.556 \| Other \| 0.785 \| 0.976 \| 0.500 \| 0.500 \| Other \| 0.887 \| 0.962 \| 0.806 \|  \| \| ICB Response \| Beck \| 0.278 \| 0.528 \| Other \| 0.121 \| 0.269 \| 0.583 \| 0.333 \| Cluster2 \| 0.245 \| 0.707 \| 0.500 \| 0.389 \| Cluster3 \| 0.887 \| 0.962 \| 0.182 \|  \| \| ICB Response \| Calabro \| 0.556 \| 0.417 \| Cluster1 \| 0.676 \| 0.850 \| 0.417 \| 0.444 \| Other \| 0.785 \| 0.976 \| 0.417 \| 0.500 \| Other \| 0.962 \| 0.962 \| 0.860 \|  \| \| ICB Response \| Ji \| 0.389 \| 0.472 \| Other \| 0.676 \| 0.850 \| 0.389 \| 0.500 \| Other \| 0.956 \| 0.976 \| 0.528 \| 0.389 \| Cluster3 \| 0.813 \| 0.962 \| 0.881 \|  \| \| ICB Response \| Kochi \| 0.667 \| 0.417 \| Cluster1 \| 0.591 \| 0.828 \| 0.194 \| 0.500 \| Other \| 0.412 \| 0.841 \| 0.472 \| 0.222 \| Cluster3 \| 0.813 \| 0.962 \| 0.610 \|  \| \| ICB Response \| Wolfe \| 0.389 \| 0.472 \| Other \| 0.953 \| 0.973 \| 0.444 \| 0.444 \| Cluster2 \| 0.956 \| 0.976 \| 0.472 \| 0.389 \| Cluster3 \| 0.887 \| 0.962 \| 0.981 \|  \| \| ICB Resistance \| Chang \| 0.222 \| 0.583 \| Other \| 0.091 \| 0.235 \| 0.694 \| 0.444 \| Cluster2 \| 0.549 \| 0.927 \| 0.528 \| 0.389 \| Cluster3 \| 0.475 \| 0.962 \| 0.205 \|  \| \| ICB Resistance \| Hugo \| 0.111 \| 0.528 \| Other \| 0.068 \| 0.207 \| 0.667 \| 0.389 \| Cluster2 \| 0.296 \| 0.763 \| 0.472 \| 0.389 \| Cluster3 \| 0.669 \| 0.962 \| 0.134 \|  \| \| ICB Resistance \| Mariathasan \| 0.111 \| 0.556 \| Other \| 0.091 \| 0.235 \| 0.833 \| 0.444 \| Cluster2 \| 0.079 \| 0.638 \| 0.472 \| 0.556 \| Other \| 0.887 \| 0.962 \| 0.079 \|  \| \| ICB Resistance \| Teschendorff \| 0.056 \| 0.583 \| Other \| 0.003 \| 0.072 \| 0.694 \| 0.444 \| Cluster2 \| 0.245 \| 0.707 \| 0.528 \| 0.389 \| Cluster3 \| 0.364 \| 0.962 \| 0.025 \| * \| \| Angiogenesis \| Gourley \| 0.167 \| 0.556 \| Other \| 0.121 \| 0.269 \| 0.611 \| 0.500 \| Cluster2 \| 0.477 \| 0.866 \| 0.556 \| 0.389 \| Cluster3 \| 0.601 \| 0.962 \| 0.243 \|  \| \| Angiogenesis \| Anders \| 0.278 \| 0.472 \| Other \| 0.953 \| 0.973 \| 0.361 \| 0.500 \| Other \| 0.477 \| 0.866 \| 0.528 \| 0.333 \| Cluster3 \| 0.475 \| 0.962 \| 0.689 \|  \| \| Angiogenesis \| Bentink \| 0.056 \| 0.583 \| Other \| 0.012 \| 0.176 \| 0.611 \| 0.444 \| Cluster2 \| 0.412 \| 0.841 \| 0.583 \| 0.389 \| Cluster3 \| 0.315 \| 0.962 \| 0.054 \|  \| \| Angiogenesis \| Mendiola \| 0.444 \| 0.528 \| Other \| 0.432 \| 0.706 \| 0.694 \| 0.389 \| Cluster2 \| 0.102 \| 0.638 \| 0.361 \| 0.611 \| Other \| 0.475 \| 0.962 \| 0.213 \|  \| \| EMT \| Knight \| 0.111 \| 0.528 \| Other \| 0.032 \| 0.176 \| 0.333 \| 0.500 \| Other \| 0.412 \| 0.841 \| 0.583 \| 0.222 \| Cluster3 \| 0.014 \| 0.665 \| 0.032 \| * \| \| EMT \| MsigDB.EMT \| 0.056 \| 0.583 \| Other \| 0.032 \| 0.176 \| 0.611 \| 0.389 \| Cluster2 \| 0.245 \| 0.707 \| 0.528 \| 0.500 \| Cluster3 \| 0.601 \| 0.962 \| 0.078 \|  \| \| EMT \| Lee \| 0.056 \| 0.583 \| Other \| 0.047 \| 0.192 \| 0.694 \| 0.389 \| Cluster2 \| 0.102 \| 0.638 \| 0.472 \| 0.500 \| Other \| 0.962 \| 0.962 \| 0.062 \|  \| \| EMT \| Rokavec \| 0.222 \| 0.528 \| Other \| 0.591 \| 0.828 \| 0.389 \| 0.556 \| Other \| 0.785 \| 0.976 \| 0.583 \| 0.278 \| Cluster3 \| 0.475 \| 0.962 \| 0.717 \|  \| \| EMT \| Wagle \| 0.222 \| 0.472 \| Other \| 0.300 \| 0.525 \| 0.139 \| 0.500 \| Other \| 0.202 \| 0.707 \| 0.556 \| 0.167 \| Cluster3 \| 0.043 \| 0.962 \| 0.122 \|  \| | |
| Significance: * Kruskal-Wallis p <0.05; ** Kruskal-Wallis p <0.01; ***Kruskal-Wallis p <0.001. |  |

| **Supplementary Table 6.** Comparison of gene signature scores between Immune High Biopsy Samples v Immune High Resection Samples | | | |
| --- | --- | --- | --- |
| Category | Signature | Mann-Whitney U p-value | FDR |
| Acquired Immune | CD45 | 0.028 | 0.084 |
| Acquired Immune | B | 0.167 | 0.255 |
| Acquired Immune | T | 1.000 | 1.000 |
| Acquired Immune | CD8 | 0.548 | 0.688 |
| Acquired Immune | Cytotoxic | 0.092 | 0.179 |
| Acquired Immune | Exhausted | 0.437 | 0.594 |
| Acquired Immune | Th1 | 0.905 | 0.964 |
| Acquired Immune | Treg | 1.000 | 1.000 |
| Innate Immune | DC | 0.095 | 0.179 |
| Innate Immune | Macrophages | 0.028 | 0.084 |
| Innate Immune | Neutrophils | 0.381 | 0.549 |
| Innate Immune | NK.CD56dim | 0.437 | 0.594 |
| Innate Immune | NK | 0.024 | 0.084 |
| Innate Immune | Mast | 0.024 | 0.084 |
| Innate Immune | M2 | 0.548 | 0.688 |
| Interferon Signalling | Complement | 0.028 | 0.084 |
| Interferon Signalling | IL2.STAT5 | 0.024 | 0.084 |
| Interferon Signalling | Inflammatory.Response | 0.024 | 0.084 |
| Interferon Signalling | IFNA | 0.028 | 0.084 |
| Interferon Signalling | IFNG | 0.052 | 0.121 |
| Interferon Signalling | TGFB | 0.024 | 0.084 |
| Immune Checkpoints | PDL1 | 0.052 | 0.121 |
| Immune Checkpoints | CD40 | 0.024 | 0.084 |
| Immune Checkpoints | CD27 | 0.714 | 0.854 |
| Immune Checkpoints | CTLA4 | 0.905 | 0.964 |
| Immune Checkpoints | LAG3 | 0.795 | 0.928 |
| Immune Checkpoints | IDO1 | 0.024 | 0.084 |
| Immune Checkpoints | HAVCR2 | 0.024 | 0.084 |
| Immune Checkpoints | PD1 | 0.167 | 0.255 |
| ICB Response | DDIR | 0.154 | 0.255 |
| ICB Response | Ayers | 0.024 | 0.084 |
| ICB Response | Beck | 0.070 | 0.155 |
| ICB Response | Calabro | 0.905 | 0.964 |
| ICB Response | Ji | 0.028 | 0.084 |
| ICB Response | Kochi | 0.092 | 0.179 |
| ICB Response | Wolfe | 0.548 | 0.688 |
| ICB Resistance | Chang | 0.262 | 0.389 |
| ICB Resistance | Hugo | 0.048 | 0.121 |
| ICB Resistance | Mariathasan | 0.167 | 0.255 |
| ICB Resistance | Teschendorff | 0.024 | 0.084 |
| Angiogenesis | Gourley | 0.905 | 0.964 |
| Angiogenesis | Anders | 0.714 | 0.854 |
| Angiogenesis | Bentink | 0.024 | 0.084 |
| Angiogenesis | Mendiola | 1.000 | 1.000 |
| EMT | Knight | 0.095 | 0.179 |
| EMT | MsigDB.EMT | 0.048 | 0.121 |
| EMT | Lee | 0.048 | 0.121 |
| EMT | Rokavec | 0.167 | 0.255 |
| EMT | Wagle | 0.167 | 0.255 |

| **Supplementary Table 7.** Comparison of gene signature scores between Immune Mixed Biopsy Samples v Immune Mixed Resection Samples | | | |
| --- | --- | --- | --- |
| Category | Signature | Mann-Whitney U p-value | FDR |
| Acquired Immune | CD45 | 0.722 | 0.902 |
| Acquired Immune | B | 0.147 | 0.654 |
| Acquired Immune | T | 0.140 | 0.654 |
| Acquired Immune | CD8 | 0.283 | 0.732 |
| Acquired Immune | Cytotoxic | 0.792 | 0.902 |
| Acquired Immune | Exhausted | 0.947 | 1.000 |
| Acquired Immune | Th1 | 0.722 | 0.902 |
| Acquired Immune | Treg | 0.598 | 0.879 |
| Innate Immune | DC | 0.381 | 0.812 |
| Innate Immune | Macrophages | 1.000 | 1.000 |
| Innate Immune | Neutrophils | 0.497 | 0.879 |
| Innate Immune | NK.CD56dim | 0.235 | 0.732 |
| Innate Immune | NK | 0.180 | 0.710 |
| Innate Immune | Mast | 0.283 | 0.732 |
| Innate Immune | M2 | 0.742 | 0.902 |
| Interferon Signalling | Complement | 0.093 | 0.654 |
| Interferon Signalling | IL2.STAT5 | 0.628 | 0.879 |
| Interferon Signalling | Inflammatory.Response | 0.582 | 0.879 |
| Interferon Signalling | IFNA | 0.539 | 0.879 |
| Interferon Signalling | IFNG | 0.674 | 0.893 |
| Interferon Signalling | TGFB | 0.418 | 0.818 |
| Immune Checkpoints | PDL1 | 0.291 | 0.732 |
| Immune Checkpoints | CD40 | 0.792 | 0.902 |
| Immune Checkpoints | CD27 | 0.456 | 0.860 |
| Immune Checkpoints | CTLA4 | 0.582 | 0.879 |
| Immune Checkpoints | LAG3 | 0.203 | 0.710 |
| Immune Checkpoints | IDO1 | 0.314 | 0.732 |
| Immune Checkpoints | HAVCR2 | 0.628 | 0.879 |
| Immune Checkpoints | PD1 | 0.674 | 0.893 |
| ICB Response | DDIR | 1.000 | 1.000 |
| ICB Response | Ayers | 0.771 | 0.902 |
| ICB Response | Beck | 0.923 | 1.000 |
| ICB Response | Calabro | 0.113 | 0.654 |
| ICB Response | Ji | 0.974 | 1.000 |
| ICB Response | Kochi | 0.510 | 0.879 |
| ICB Response | Wolfe | 0.821 | 0.915 |
| ICB Resistance | Chang | 0.107 | 0.654 |
| ICB Resistance | Hugo | 0.203 | 0.710 |
| ICB Resistance | Mariathasan | 0.093 | 0.654 |
| ICB Resistance | Teschendorff | 0.043 | 0.654 |
| Angiogenesis | Gourley | 0.628 | 0.879 |
| Angiogenesis | Anders | 0.418 | 0.818 |
| Angiogenesis | Bentink | 0.107 | 0.654 |
| Angiogenesis | Mendiola | 0.381 | 0.812 |
| EMT | Knight | 0.283 | 0.732 |
| EMT | MsigDB.EMT | 0.140 | 0.654 |
| EMT | Lee | 0.314 | 0.732 |
| EMT | Rokavec | 0.140 | 0.654 |
| EMT | Wagle | 0.123 | 0.654 |

| **Supplementary Table 8.** Comparison of gene signature scores between Immune Low Biopsy Samples v Immune Low Resection Samples | | | |
| --- | --- | --- | --- |
| Category | Signature | Mann-Whitney U p-value | FDR |
| Acquired Immune | CD45 | 0.230 | 0.940 |
| Acquired Immune | B | 0.185 | 0.940 |
| Acquired Immune | T | 0.230 | 0.940 |
| Acquired Immune | CD8 | 0.570 | 1.000 |
| Acquired Immune | Cytotoxic | 0.788 | 1.000 |
| Acquired Immune | Exhausted | 1.000 | 1.000 |
| Acquired Immune | Th1 | 0.412 | 1.000 |
| Acquired Immune | Treg | 0.449 | 1.000 |
| Innate Immune | DC | 0.648 | 1.000 |
| Innate Immune | Macrophages | 0.073 | 0.891 |
| Innate Immune | Neutrophils | 0.850 | 1.000 |
| Innate Immune | NK.CD56dim | 0.527 | 1.000 |
| Innate Immune | NK | 0.705 | 1.000 |
| Innate Immune | Mast | 0.164 | 0.940 |
| Innate Immune | M2 | 1.000 | 1.000 |
| Interferon Signalling | Complement | 0.927 | 1.000 |
| Interferon Signalling | IL2.STAT5 | 0.109 | 0.940 |
| Interferon Signalling | Inflammatory.Response | 0.927 | 1.000 |
| Interferon Signalling | IFNA | 0.648 | 1.000 |
| Interferon Signalling | IFNG | 0.648 | 1.000 |
| Interferon Signalling | TGFB | 0.527 | 1.000 |
| Immune Checkpoints | PDL1 | 0.412 | 1.000 |
| Immune Checkpoints | CD40 | 0.012 | 0.594 |
| Immune Checkpoints | CD27 | 0.412 | 1.000 |
| Immune Checkpoints | CTLA4 | 1.000 | 1.000 |
| Immune Checkpoints | LAG3 | 0.570 | 1.000 |
| Immune Checkpoints | IDO1 | 0.705 | 1.000 |
| Immune Checkpoints | HAVCR2 | 0.927 | 1.000 |
| Immune Checkpoints | PD1 | 1.000 | 1.000 |
| ICB Response | DDIR | 0.648 | 1.000 |
| ICB Response | Ayers | 0.648 | 1.000 |
| ICB Response | Beck | 0.042 | 0.891 |
| ICB Response | Calabro | 0.185 | 0.940 |
| ICB Response | Ji | 0.230 | 0.940 |
| ICB Response | Kochi | 0.527 | 1.000 |
| ICB Response | Wolfe | 1.000 | 1.000 |
| ICB Resistance | Chang | 0.412 | 1.000 |
| ICB Resistance | Hugo | 0.927 | 1.000 |
| ICB Resistance | Mariathasan | 0.850 | 1.000 |
| ICB Resistance | Teschendorff | 0.788 | 1.000 |
| Angiogenesis | Gourley | 0.788 | 1.000 |
| Angiogenesis | Anders | 0.344 | 1.000 |
| Angiogenesis | Bentink | 0.927 | 1.000 |
| Angiogenesis | Mendiola | 0.073 | 0.891 |
| EMT | Knight | 0.527 | 1.000 |
| EMT | MsigDB.EMT | 0.927 | 1.000 |
| EMT | Lee | 0.315 | 1.000 |
| EMT | Rokavec | 0.705 | 1.000 |
| EMT | Wagle | 0.230 | 0.940 |

**Supplementary Table 9.** Gene signatures which underwent a significant change and were common across both Xelox only and Xelox plus AZD8931 treatments, as determined by Wilcoxon Signed Rank testing.

| **Hallmark** | **Signature** | **Signature as named in clara^T^ report** | **Score Increase or Decrease Pre to Post Treatment**  **(Xelox Only)** | **Score Increase or Decrease Pre to Post Treatment (Xelox+AZD8931)** | **Consensus** |
| --- | --- | --- | --- | --- | --- |
| Angiogenesis | Almac_Angio_Assay | Almac Angio Assay | Increased | Increased | Yes |
| Angiogenesis | Hu_et_al | Angiogenesis Signature A | Increased | Increased | Yes |
| EMT | Becht_Fibroblasts | MCPCounter-Fibroblasts | Increased | Increased | Yes |
| EMT | Lee_et_al | Mesenchymal Subtype | Increased | Increased | Yes |
| EMT | Mak_et_al | Pan-Can EMT Signature B | Increased | Increased | Yes |
| Energetics | HALLMARK_MYOGENESIS | NA | Increased | Increased | Yes |
| Energetics | HALLMARK_SPERMATOGENESIS | NA | Decreased | Decreased | Yes |
| Energetics | VonRundstedt_et_al | Metabolic Pathway Signature | Decreased | Decreased | Yes |
| Genome Instability | Almac_DNA_Damage_Assay | Almac DNA Damage Assay | Decreased | Decreased | Yes |
| Genome Instability | Peng_et_al | HRD Gene Signature | Decreased | Decreased | Yes |
| Genome Instability | Severson_et_al | BRCA1ness Signature | Decreased | Decreased | Yes |
| Immuno-Oncology | Almac_IO_Assay | Almac I-O Assay | Decreased | Decreased | Yes |
| Immuno-Oncology | Prat_Macrophages...140 | NA | Increased | Increased | Yes |
| Inflammation | Prat_Macrophages...177 | Immune Response (Macrophages) | Increased | Increased | Yes |

**Supplementary Table 10.** Gene signatures which underwent a significant change when assessing n=6 paired patient biopsy and resection specimens where Xelox Only treatment was received, as determined by Wilcoxon Signed Rank testing. Listed signatures changes are unique to Xelox Only treatment.

| **Hallmark** | **Signature** | **Signature as named in clara^T^ report** | **Wilcoxon Signed Rank p-value**  **(Before v After)** | **FDR**  **(Before v After)** | **Score Increase or Decrease Pre to Post Treatment** |
| --- | --- | --- | --- | --- | --- |
| Angiogenesis | HALLMARK_COAGULATION | NA | 0.031 | 0.179 | Increased |
| Angiogenesis | HALLMARK_HYPOXIA | NA | 0.031 | 0.179 | Increased |
| Cell Death | HALLMARK_APOPTOSIS | Apoptosis Enrichment Score | 0.031 | 0.179 | Increased |
| EMT | HALLMARK_EPITHELIAL_MESENCHYMAL_TRANSITION | EMT Enrichment Score | 0.031 | 0.179 | Increased |
| Energetics | Daemen_et_al | GLS1/GCS Co-Dependency GES | 0.031 | 0.179 | Increased |
| Energetics | HALLMARK_ADIPOGENESIS | NA | 0.031 | 0.179 | Increased |
| Genome Instability | HALLMARK_UV_RESPONSE_DN | NA | 0.031 | 0.179 | Increased |
| Genome Instability | HALLMARK_UV_RESPONSE_UP | NA | 0.031 | 0.179 | Increased |
| Immortality | Marques_et_al | Molecular Senescence Signature | 0.031 | 0.179 | Increased |
| Immortality | Wu_et_al2 | Candidate Senescence Signature | 0.031 | 0.179 | Increased |
| Immuno-Oncology | HALLMARK_IL2_STAT5_SIGNALING | NA | 0.031 | 0.179 | Increased |
| Immuno-Oncology | HALLMARK_TGF_BETA_SIGNALING | NA | 0.031 | 0.179 | Increased |
| Immuno-Oncology | Ji_et_al | CTLA4 Response Signature | 0.031 | 0.179 | Increased |
| Immuno-Oncology | Prat_Cluster4_IFNactivation | NA | 0.031 | 0.179 | Decreased |
| Immuno-Oncology | Prat_DC | NA | 0.031 | 0.179 | Increased |
| Immuno-Oncology | Prat_Normal_mucosa | NA | 0.031 | 0.179 | Increased |
| Inflammation | HALLMARK_IL2_STAT5_SIGNALING | NA | 0.031 | 0.179 | Increased |
| Inflammation | Prat_Cluster4_IFNactivation | NA | 0.031 | 0.179 | Decreased |
| Inflammation | Prat_DC | NA | 0.031 | 0.179 | Increased |
| Inflammation | Prat_Normal_mucosa | NA | 0.031 | 0.179 | Increased |
| Proliferation | HALLMARK_TGF_BETA_SIGNALING | NA | 0.031 | 0.179 | Increased |
| Proliferation | HALLMARK_ANDROGEN_RESPONSE | NA | 0.031 | 0.179 | Increased |
| Proliferation | Sonnenblick_et_al | pSTAT3-GS Score | 0.031 | 0.179 | Increased |

**Supplementary Table 11.** Gene signatures which underwent a significant change when assessing n=11 paired patient biopsy and resection specimens where Xelox plus AZD8931 treatment was received, as determined by Wilcoxon Signed Rank testing. Listed signatures changes are unique to Xelox plus AZD8931 treatment.

| **Hallmark** | **Signature** | **Signature as named in clara^T^ report** | **Wilcoxon Signed Rank p-value**  **(Before v After)** | **FDR**  **(Before v After)** | **Score Increase or Decrease Pre to Post Treatment** |
| --- | --- | --- | --- | --- | --- |
| Angiogenesis | Anders_et_al | Global Angio Signature [33]* | 0.032 | 0.124 | Decreased |
| Angiogenesis | Mendiola_et_al | Angio Predictive G model [35] | 0.042 | 0.137 | Increased |
| Cell Death | Bredholt_et_al | Tumour Necrosis Signature [53] | 0.003 | 0.076 | Decreased |
| Cell Death | Chen_et_al | TRAIL Sensitivity Predictor [51] | 0.007 | 0.076 | Decreased |
| Cell Death | An_et_al | Autophagy-related Risk Score [55] | 0.024 | 0.110 | Increased |
| EMT | Becht_Neutrophils | NA | 0.002 | 0.076 | Decreased |
| EMT | Becht_Monocytic lineage | NA | 0.014 | 0.097 | Decreased |
| EMT | Tan_et_al | EMT Signature Estimate [21] | 0.014 | 0.097 | Decreased |
| EMT | Rokavec_et_al | Pan-Can EMT Signature A [22] | 0.019 | 0.109 | Decreased |
| EMT | HALLMARK_APICAL_JUNCTION | NA | 0.042 | 0.137 | Increased |
| Energetics | Haider_et_al | Core Metabolic Signature [65]* | 0.002 | 0.076 | Decreased |
| Energetics | HALLMARK_FATTY_ACID_METABOLISM | NA | 0.007 | 0.076 | Decreased |
| Energetics | HALLMARK_BILE_ACID_METABOLISM | NA | 0.010 | 0.083 | Decreased |
| Energetics | Chen_et_al2 | Glycolytic GE Signature Score [63] | 0.024 | 0.110 | Decreased |
| Energetics | HALLMARK_PEROXISOME | NA | 0.024 | 0.110 | Decreased |
| Energetics | HALLMARK_XENOBIOTIC_METABOLISM | NA | 0.024 | 0.110 | Decreased |
| Energetics | HALLMARK_GLYCOLYSIS | NA | 0.032 | 0.124 | Decreased |
| Evading Growth | Mizuno_et_al | Cell Cycle Signature (CCS) [69] | 0.010 | 0.083 | Decreased |
| Evading Growth | Locatelli_et_al | E2F Gene Signature [73]* | 0.019 | 0.109 | Decreased |
| Evading Growth | HALLMARK_G2M_CHECKPOINT | G2M Checkpoint Enrichment [16]* | 0.032 | 0.124 | Decreased |
| Evading Growth | Chen_et_al3 | RB1-loss Signature (RBS) [72]* | 0.042 | 0.137 | Increased |
| Genome Instability | Konstantinopoulos_et_al | BRCAness Profile [37] | 0.007 | 0.076 | Decreased |
| Genome Instability | Carter_et_al | CIN25 Signature [40] | 0.010 | 0.083 | Decreased |
| Genome Instability | Szasz_et_al | CIN4 Signature [41] | 0.024 | 0.110 | Decreased |
| Genome Instability | Kang_et_al | DDR Pathway Focused Score [42]* | 0.042 | 0.137 | Decreased |
| Immortality | Purcell_et_al | Senescence-associated Signature [77]* | 0.001 | 0.076 | Decreased |
| Immortality | Hernandez-Segura_et_al | Universal Senescence Signature [74]* | 0.042 | 0.137 | Decreased |
| Immuno-Oncology | HALLMARK_INTERFERON_ALPHA_RESPONSE | NA | 0.007 | 0.076 | Decreased |
| Immuno-Oncology | Prat_Neutrophils | NA | 0.007 | 0.076 | Decreased |
| Immuno-Oncology | Wolf_et_al | TCGA IFN gamma Signature [10]* | 0.007 | 0.076 | Decreased |
| Immuno-Oncology | Kochi_et_al | TILs-related GS [4] | 0.010 | 0.083 | Decreased |
| Immuno-Oncology | Prat_Mast_cells | NA | 0.010 | 0.083 | Increased |
| Immuno-Oncology | HALLMARK_COMPLEMENT | NA | 0.014 | 0.097 | Decreased |
| Immuno-Oncology | HALLMARK_INTERFERON_GAMMA_RESPONSE | Immune Enrichment Score [15,16]* | 0.019 | 0.109 | Decreased |
| Immuno-Oncology | Danaher_Neutrophils | NA | 0.024 | 0.110 | Decreased |
| Immuno-Oncology | Danaher_Macrophages | NA | 0.032 | 0.124 | Increased |
| Immuno-Oncology | HALLMARK_INFLAMMATORY_RESPONSE | NA | 0.042 | 0.137 | Decreased |
| Inflammation | Wu_et_al | IL1-beta Signature [61]* | 0.002 | 0.076 | Decreased |
| Inflammation | Prat_Neutrophils | NA | 0.007 | 0.076 | Decreased |
| Inflammation | Prat_Mast_cells | NA | 0.010 | 0.083 | Increased |
| Inflammation | Hernandez_et_al | IKK-beta Signature [58]* | 0.019 | 0.109 | Increased |
| Inflammation | Danaher_Neutrophils | NA | 0.024 | 0.110 | Decreased |
| Inflammation | Danaher_Macrophages | TILs Macrophages [3]* | 0.032 | 0.124 | Increased |
| Inflammation | HALLMARK_INFLAMMATORY_RESPONSE | Inflammatory Response Enrichment [16]* | 0.042 | 0.137 | Decreased |
| Inflammation | HALLMARK_ALLOGRAFT_REJECTION | NA | 0.042 | 0.137 | Decreased |
| Inflammation | HALLMARK_IL6_JAK_STAT3_SIGNALING | NA | 0.042 | 0.137 | Decreased |
| Proliferation | Balko_et_al_SensitivityScore | EGFR Sensitivity Signature [47]* | 0.007 | 0.076 | Decreased |
| Proliferation | Bertucci_et_al | ERBB2-specific GES [44]* | 0.019 | 0.109 | Decreased |
| Proliferation | Singh_et_al | KRAS Dependency Signature [48]* | 0.019 | 0.109 | Decreased |
| Proliferation | HALLMARK_PI3K_AKT_MTOR_SIGNALING | PI3K/AKT Enrichment Score [16]* | 0.032 | 0.124 | Decreased |
| Proliferation | HALLMARK_MTORC1_SIGNALING | NA | 0.042 | 0.137 | Decreased |

**Supplementary Table 12.** Biological processes enriched in the Responders (Mandard Score 1-2) relative to Non-Responders (Mandard Score 3-5) to neo-adjuvant therapy following Gene Set Enrichment Analysis with the KEGG and REACTOME gene sets (limited to nominal p value <0.05, FDR <0.2).

| NAME | SIZE | ES | NES | NOM p-val | FDR q-val | FWER p-val |
| --- | --- | --- | --- | --- | --- | --- |
| REACTOME_INTERLEUKIN_10_SIGNALING | 42 | -0.720742 | -2.5605073 | 0 | 0 | 0 |
| KEGG_LEISHMANIA_INFECTION | 68 | -0.6524913 | -2.5271337 | 0 | 0 | 0 |
| REACTOME_INTERLEUKIN_4_AND_INTERLEUKIN_13_SIGNALING | 108 | -0.5713342 | -2.4138482 | 0 | 0 | 0 |
| KEGG_CYTOKINE_CYTOKINE_RECEPTOR_INTERACTION | 243 | -0.5009049 | -2.3357835 | 0 | 0 | 0 |
| KEGG_GRAFT_VERSUS_HOST_DISEASE | 34 | -0.6780767 | -2.2855768 | 0 | 1.59E-05 | 0.001 |
| REACTOME_CHEMOKINE_RECEPTORS_BIND_CHEMOKINES | 49 | -0.6186224 | -2.2167687 | 0 | 9.84E-05 | 0.009 |
| REACTOME_NEUTROPHIL_DEGRANULATION | 446 | -0.4444633 | -2.1874695 | 0 | 1.01E-04 | 0.01 |
| KEGG_ALLOGRAFT_REJECTION | 34 | -0.6278971 | -2.1354947 | 0 | 2.15E-04 | 0.025 |
| KEGG_TOLL_LIKE_RECEPTOR_SIGNALING_PATHWAY | 98 | -0.5161927 | -2.1167755 | 0 | 3.44E-04 | 0.043 |
| REACTOME_INTERFERON_ALPHA_BETA_SIGNALING | 71 | -0.5424747 | -2.1072588 | 0 | 3.88E-04 | 0.05 |
| REACTOME_INTERLEUKIN_12_FAMILY_SIGNALING | 53 | -0.5677685 | -2.09924 | 0 | 4.65E-04 | 0.062 |
| KEGG_TYPE_I_DIABETES_MELLITUS | 40 | -0.6013305 | -2.0947497 | 0 | 5.03E-04 | 0.067 |
| REACTOME_SIGNALING_BY_INTERLEUKINS | 440 | -0.4211366 | -2.0696354 | 0 | 7.58E-04 | 0.106 |
| REACTOME_GENE_AND_PROTEIN_EXPRESSION_BY_JAK_STAT_SIGNALING_AFTER_INTERLEUKIN_12_STIMULATION | 34 | -0.6123757 | -2.0306287 | 0 | 0.00116503 | 0.176 |
| KEGG_NOD_LIKE_RECEPTOR_SIGNALING_PATHWAY | 58 | -0.541469 | -2.0251484 | 0 | 0.00116277 | 0.181 |
| KEGG_HEMATOPOIETIC_CELL_LINEAGE | 83 | -0.4896665 | -1.9936082 | 0 | 0.00164303 | 0.27 |
| KEGG_SYSTEMIC_LUPUS_ERYTHEMATOSUS | 52 | -0.5333282 | -1.9585507 | 0 | 0.00254584 | 0.423 |
| REACTOME_ACTIVATION_OF_MATRIX_METALLOPROTEINASES | 30 | -0.596315 | -1.9502292 | 0 | 0.00279576 | 0.462 |
| REACTOME_INTERLEUKIN_12_SIGNALING | 43 | -0.5636086 | -1.9445217 | 0 | 0.00297068 | 0.483 |
| KEGG_ANTIGEN_PROCESSING_AND_PRESENTATION | 75 | -0.4859686 | -1.9325819 | 0 | 0.00343526 | 0.535 |
| REACTOME_IMMUNOREGULATORY_INTERACTIONS_BETWEEN_A_LYMPHOID_AND_A_NON_LYMPHOID_CELL | 127 | -0.4469712 | -1.911904 | 0 | 0.00424988 | 0.649 |
| REACTOME_ROS_AND_RNS_PRODUCTION_IN_PHAGOCYTES | 34 | -0.5617712 | -1.9103404 | 0 | 0.00427565 | 0.654 |
| REACTOME_NGF_STIMULATED_TRANSCRIPTION | 39 | -0.5384783 | -1.8991026 | 0 | 0.0047837 | 0.72 |
| KEGG_ASTHMA | 26 | -0.6008968 | -1.8973656 | 0.00344234 | 0.00485411 | 0.725 |
| REACTOME_PEPTIDE_LIGAND_BINDING_RECEPTORS | 179 | -0.4195401 | -1.8932825 | 0 | 0.00509544 | 0.742 |
| REACTOME_INTERFERON_GAMMA_SIGNALING | 89 | -0.4570719 | -1.8833188 | 0 | 0.00547335 | 0.79 |
| KEGG_JAK_STAT_SIGNALING_PATHWAY | 147 | -0.4326954 | -1.8821456 | 0 | 0.00559978 | 0.799 |
| REACTOME_IRON_UPTAKE_AND_TRANSPORT | 57 | -0.5046814 | -1.8786526 | 0 | 0.00585453 | 0.813 |
| KEGG_AUTOIMMUNE_THYROID_DISEASE | 49 | -0.5168012 | -1.8697428 | 0 | 0.00635504 | 0.843 |
| REACTOME_CLASS_A_1_RHODOPSIN_LIKE_RECEPTORS | 298 | -0.386303 | -1.8677189 | 0 | 0.00647826 | 0.847 |
| REACTOME_RESPONSE_TO_ELEVATED_PLATELET_CYTOSOLIC_CA2 | 123 | -0.4349742 | -1.8662596 | 0 | 0.00656956 | 0.854 |
| KEGG_NATURAL_KILLER_CELL_MEDIATED_CYTOTOXICITY | 121 | -0.4381699 | -1.858955 | 0 | 0.00707332 | 0.877 |
| REACTOME_REGULATION_OF_IFNA_IFNB_SIGNALING | 26 | -0.5914127 | -1.8468548 | 0 | 0.00798199 | 0.899 |
| REACTOME_PURINERGIC_SIGNALING_IN_LEISHMANIASIS_INFECTION | 26 | -0.6014649 | -1.8439472 | 0.00163934 | 0.00833813 | 0.909 |
| REACTOME_SCAVENGING_BY_CLASS_A_RECEPTORS | 19 | -0.6140367 | -1.8412194 | 0.00537634 | 0.00853069 | 0.915 |
| KEGG_CHEMOKINE_SIGNALING_PATHWAY | 174 | -0.4053221 | -1.8173989 | 0 | 0.01033127 | 0.968 |
| REACTOME_INSULIN_RECEPTOR_RECYCLING | 30 | -0.5498784 | -1.7914076 | 0.00166667 | 0.01305022 | 0.989 |
| KEGG_APOPTOSIS | 83 | -0.4400862 | -1.7795196 | 0 | 0.01439325 | 0.993 |
| REACTOME_BIOSYNTHESIS_OF_SPECIALIZED_PRORESOLVING_MEDIATORS_SPMS | 18 | -0.6236559 | -1.7766491 | 0.00892857 | 0.0147879 | 0.993 |
| REACTOME_INTERLEUKIN_20_FAMILY_SIGNALING | 22 | -0.5824841 | -1.7709934 | 0.00670017 | 0.01559392 | 0.996 |
| REACTOME_FCGR_ACTIVATION | 19 | -0.6056479 | -1.7680664 | 0.00874126 | 0.01598567 | 0.997 |
| REACTOME_INACTIVATION_OF_CSF3_G_CSF_SIGNALING | 22 | -0.5857089 | -1.7513818 | 0.00679117 | 0.01825267 | 0.999 |
| REACTOME_BINDING_AND_UPTAKE_OF_LIGANDS_BY_SCAVENGER_RECEPTORS | 46 | -0.485192 | -1.7412187 | 0 | 0.01991278 | 0.999 |
| REACTOME_TNFS_BIND_THEIR_PHYSIOLOGICAL_RECEPTORS | 24 | -0.5577978 | -1.7298473 | 0.00816994 | 0.02157753 | 0.999 |
| KEGG_RIG_I_LIKE_RECEPTOR_SIGNALING_PATHWAY | 67 | -0.4435567 | -1.7254137 | 0.0015456 | 0.02247681 | 0.999 |
| REACTOME_INTERFERON_SIGNALING | 190 | -0.3788275 | -1.7138832 | 0 | 0.024739 | 1 |
| REACTOME_TRANSFERRIN_ENDOCYTOSIS_AND_RECYCLING | 31 | -0.5194592 | -1.7112256 | 0.00689655 | 0.02523649 | 1 |
| REACTOME_NUCLEAR_EVENTS_KINASE_AND_TRANSCRIPTION_FACTOR_ACTIVATION | 61 | -0.4490678 | -1.706396 | 0.00325203 | 0.02609431 | 1 |
| REACTOME_LEISHMANIA_INFECTION | 167 | -0.3834747 | -1.705984 | 0 | 0.0260276 | 1 |
| REACTOME_MYD88_INDEPENDENT_TLR4_CASCADE | 106 | -0.405845 | -1.6878182 | 0.0014771 | 0.02939307 | 1 |
| REACTOME_TOLL_LIKE_RECEPTOR_9_TLR9_CASCADE | 102 | -0.4004719 | -1.6818442 | 0.00156495 | 0.03082139 | 1 |
| KEGG_MAPK_SIGNALING_PATHWAY | 258 | -0.3551286 | -1.6766313 | 0 | 0.03173985 | 1 |
| REACTOME_SIGNALING_BY_CSF3_G_CSF | 27 | -0.5219981 | -1.6721109 | 0.00720721 | 0.03269971 | 1 |
| REACTOME_RAF_INDEPENDENT_MAPK1_3_ACTIVATION | 23 | -0.5446479 | -1.6689211 | 0.01839465 | 0.03332361 | 1 |
| REACTOME_DECTIN_2_FAMILY | 25 | -0.5289205 | -1.6668645 | 0.0152027 | 0.03377055 | 1 |
| REACTOME_TOLL_LIKE_RECEPTOR_CASCADES | 159 | -0.3785151 | -1.6658638 | 0.00144928 | 0.03385639 | 1 |
| REACTOME_FOXO_MEDIATED_TRANSCRIPTION_OF_OXIDATIVE_STRESS_METABOLIC_AND_NEURONAL_GENES | 29 | -0.5059218 | -1.6567817 | 0.0147541 | 0.03616388 | 1 |
| REACTOME_GPCR_LIGAND_BINDING | 421 | -0.33719 | -1.6540257 | 0 | 0.03696842 | 1 |
| REACTOME_PYROPTOSIS | 25 | -0.5188643 | -1.6511424 | 0.01658375 | 0.03772226 | 1 |
| REACTOME_IRAK4_DEFICIENCY_TLR2_4 | 18 | -0.5710302 | -1.6478643 | 0.01618705 | 0.03848221 | 1 |
| REACTOME_NUCLEAR_EVENTS_STIMULATED_BY_ALK_SIGNALING_IN_CANCER | 18 | -0.5625481 | -1.6344305 | 0.03141361 | 0.04211311 | 1 |
| REACTOME_FATTY_ACYL_COA_BIOSYNTHESIS | 32 | -0.4936488 | -1.6335087 | 0.00699301 | 0.04227344 | 1 |
| KEGG_REGULATION_OF_AUTOPHAGY | 33 | -0.4873558 | -1.6302093 | 0.02356902 | 0.04336779 | 1 |
| REACTOME_TOLL_LIKE_RECEPTOR_TLR1_TLR2_CASCADE | 114 | -0.3856582 | -1.6239573 | 0.00151057 | 0.04507197 | 1 |
| REACTOME_PLATELET_ACTIVATION_SIGNALING_AND_AGGREGATION | 246 | -0.3480415 | -1.6210268 | 0 | 0.04615899 | 1 |
| REACTOME_ACTIVATION_OF_IRF3_IRF7_MEDIATED_BY_TBK1_IKK_EPSILON | 15 | -0.5888186 | -1.6175191 | 0.03085299 | 0.04708475 | 1 |
| KEGG_EPITHELIAL_CELL_SIGNALING_IN_HELICOBACTER_PYLORI_INFECTION | 67 | -0.4192504 | -1.6173832 | 0 | 0.04705967 | 1 |
| KEGG_CYTOSOLIC_DNA_SENSING_PATHWAY | 51 | -0.4427213 | -1.6084969 | 0.00949367 | 0.04995103 | 1 |
| REACTOME_GRB2_SOS_PROVIDES_LINKAGE_TO_MAPK_SIGNALING_FOR_INTEGRINS | 15 | -0.5885348 | -1.6076193 | 0.02816901 | 0.04999531 | 1 |
| KEGG_NICOTINATE_AND_NICOTINAMIDE_METABOLISM | 22 | -0.5281399 | -1.6016171 | 0.02033898 | 0.05162734 | 1 |
| REACTOME_NUCLEOTIDE_BINDING_DOMAIN_LEUCINE_RICH_REPEAT_CONTAINING_RECEPTOR_NLR_SIGNALING_PATHWAYS | 55 | -0.4256235 | -1.6001607 | 0.00470219 | 0.05213761 | 1 |
| REACTOME_MITOPHAGY | 24 | -0.5241782 | -1.5974895 | 0.0147541 | 0.05263019 | 1 |
| KEGG_PRION_DISEASES | 35 | -0.4752675 | -1.5934575 | 0.01692047 | 0.05383463 | 1 |
| REACTOME_PARASITE_INFECTION | 66 | -0.4053392 | -1.5854714 | 0.0115894 | 0.05680197 | 1 |
| REACTOME_ESTROGEN_DEPENDENT_NUCLEAR_EVENTS_DOWNSTREAM_OF_ESR_MEMBRANE_SIGNALING | 23 | -0.508623 | -1.583744 | 0.03741496 | 0.05715451 | 1 |
| REACTOME_TNFR1_INDUCED_PROAPOPTOTIC_SIGNALING | 24 | -0.5089134 | -1.5819774 | 0.02159469 | 0.05791191 | 1 |
| REACTOME_DISEASES_OF_IMMUNE_SYSTEM | 31 | -0.4826817 | -1.5803007 | 0.02590674 | 0.05845381 | 1 |
| REACTOME_GPVI_MEDIATED_ACTIVATION_CASCADE | 32 | -0.4780122 | -1.5800881 | 0.02233677 | 0.05847199 | 1 |
| KEGG_VIBRIO_CHOLERAE_INFECTION | 53 | -0.4278835 | -1.5794865 | 0.0128 | 0.05864033 | 1 |
| REACTOME_NOREPINEPHRINE_NEUROTRANSMITTER_RELEASE_CYCLE | 17 | -0.5532047 | -1.5735577 | 0.02659575 | 0.06063221 | 1 |
| REACTOME_ATTENUATION_PHASE | 26 | -0.4958169 | -1.5699722 | 0.03005008 | 0.0615232 | 1 |
| KEGG_ADIPOCYTOKINE_SIGNALING_PATHWAY | 65 | -0.4041283 | -1.5679414 | 0.00816994 | 0.06229042 | 1 |
| REACTOME_CHAPERONE_MEDIATED_AUTOPHAGY | 21 | -0.5213142 | -1.5654974 | 0.04220184 | 0.06328189 | 1 |
| KEGG_CELL_ADHESION_MOLECULES_CAMS | 125 | -0.3576931 | -1.5557421 | 0.00907716 | 0.06780069 | 1 |
| REACTOME_TRANSPORT_OF_BILE_SALTS_AND_ORGANIC_ACIDS_METAL_IONS_AND_AMINE_COMPOUNDS | 83 | -0.3866588 | -1.5524461 | 0.01259843 | 0.06928964 | 1 |
| REACTOME_LATE_ENDOSOMAL_MICROAUTOPHAGY | 30 | -0.4651661 | -1.5498959 | 0.0318258 | 0.07036824 | 1 |
| REACTOME_G_ALPHA_I_SIGNALLING_EVENTS | 293 | -0.3236102 | -1.5462186 | 0 | 0.07204632 | 1 |
| KEGG_INTESTINAL_IMMUNE_NETWORK_FOR_IGA_PRODUCTION | 43 | -0.435102 | -1.5379573 | 0.01779935 | 0.07582019 | 1 |
| REACTOME_CD28_CO_STIMULATION | 33 | -0.4611705 | -1.5352843 | 0.0231405 | 0.07668686 | 1 |
| REACTOME_THE_NLRP3_INFLAMMASOME | 16 | -0.5408019 | -1.5294126 | 0.03339192 | 0.07979987 | 1 |
| REACTOME_FCGR3A_MEDIATED_IL10_SYNTHESIS | 45 | -0.4271898 | -1.5290267 | 0.02644628 | 0.07998145 | 1 |
| REACTOME_COSTIMULATION_BY_THE_CD28_FAMILY | 68 | -0.3958634 | -1.5287349 | 0.0097561 | 0.08005854 | 1 |
| REACTOME_NEGATIVE_REGULATION_OF_FGFR4_SIGNALING | 30 | -0.4620315 | -1.5246199 | 0.04529617 | 0.08216428 | 1 |
| REACTOME_GLUTAMATE_NEUROTRANSMITTER_RELEASE_CYCLE | 23 | -0.4932077 | -1.5217065 | 0.04035088 | 0.08323608 | 1 |
| REACTOME_SIGNALING_BY_PTK6 | 53 | -0.4133461 | -1.518242 | 0.02580645 | 0.08528545 | 1 |
| REACTOME_ANTIGEN_PRESENTATION_FOLDING_ASSEMBLY_AND_PEPTIDE_LOADING_OF_CLASS_I_MHC | 29 | -0.4642221 | -1.5168593 | 0.03161398 | 0.08602803 | 1 |
| REACTOME_DEGRADATION_OF_THE_EXTRACELLULAR_MATRIX | 132 | -0.3537851 | -1.516707 | 0.00285714 | 0.08603191 | 1 |
| REACTOME_RHO_GTPASES_ACTIVATE_WASPS_AND_WAVES | 36 | -0.4448638 | -1.5134959 | 0.03525641 | 0.08730269 | 1 |
| REACTOME_REGULATED_NECROSIS | 54 | -0.4068946 | -1.5081632 | 0.0192 | 0.09022111 | 1 |
| REACTOME_REGULATION_OF_TLR_BY_ENDOGENOUS_LIGAND | 20 | -0.5085951 | -1.5081033 | 0.03367003 | 0.09016123 | 1 |
| REACTOME_SYNTHESIS_OF_VERY_LONG_CHAIN_FATTY_ACYL_COAS | 19 | -0.5130464 | -1.5078682 | 0.03872054 | 0.09000833 | 1 |
| REACTOME_SELECTIVE_AUTOPHAGY | 70 | -0.3857251 | -1.5062428 | 0.01567398 | 0.0906168 | 1 |
| KEGG_NEUROACTIVE_LIGAND_RECEPTOR_INTERACTION | 260 | -0.3184661 | -1.5001236 | 0 | 0.09367377 | 1 |
| REACTOME_NEGATIVE_REGULATION_OF_FGFR3_SIGNALING | 28 | -0.4661931 | -1.4964619 | 0.04026846 | 0.0955136 | 1 |
| REACTOME_ION_CHANNEL_TRANSPORT | 178 | -0.3322842 | -1.4960707 | 0.00431035 | 0.09546816 | 1 |
| REACTOME_ZINC_TRANSPORTERS | 15 | -0.5381506 | -1.4869539 | 0.03677758 | 0.10079857 | 1 |
| REACTOME_INTERLEUKIN_3_INTERLEUKIN_5_AND_GM_CSF_SIGNALING | 47 | -0.4137798 | -1.4836456 | 0.03610675 | 0.10243268 | 1 |
| REACTOME_SARS_COV_1_ACTIVATES_MODULATES_INNATE_IMMUNE_RESPONSES | 34 | -0.4328067 | -1.478971 | 0.03304348 | 0.1050117 | 1 |
| REACTOME_PLATELET_AGGREGATION_PLUG_FORMATION | 37 | -0.424956 | -1.465427 | 0.04390244 | 0.11370992 | 1 |
| REACTOME_NEUROTRANSMITTER_RELEASE_CYCLE | 49 | -0.4030514 | -1.4612461 | 0.0375817 | 0.11606167 | 1 |
| REACTOME_COLLAGEN_DEGRADATION | 60 | -0.3817584 | -1.4609308 | 0.02145215 | 0.11598889 | 1 |
| REACTOME_EXTRA_NUCLEAR_ESTROGEN_SIGNALING | 73 | -0.3749023 | -1.4598132 | 0.02689873 | 0.11605609 | 1 |
| REACTOME_ECM_PROTEOGLYCANS | 76 | -0.368086 | -1.4523712 | 0.02469136 | 0.12038817 | 1 |
| REACTOME_CELL_SURFACE_INTERACTIONS_AT_THE_VASCULAR_WALL | 135 | -0.3385046 | -1.4484063 | 0.01043219 | 0.12287359 | 1 |
| REACTOME_DETOXIFICATION_OF_REACTIVE_OXYGEN_SPECIES | 34 | -0.4236814 | -1.4479432 | 0.04304636 | 0.12300398 | 1 |
| REACTOME_NEGATIVE_REGULATION_OF_THE_PI3K_AKT_NETWORK | 111 | -0.3441373 | -1.4397458 | 0.02184087 | 0.12864935 | 1 |
| KEGG_T_CELL_RECEPTOR_SIGNALING_PATHWAY | 104 | -0.3422044 | -1.4350241 | 0.02105263 | 0.13177688 | 1 |
| KEGG_LYSOSOME | 118 | -0.3363904 | -1.4339069 | 0.0075643 | 0.13256072 | 1 |
| REACTOME_CONSTITUTIVE_SIGNALING_BY_ABERRANT_PI3K_IN_CANCER | 76 | -0.3635962 | -1.4335972 | 0.03139718 | 0.1326943 | 1 |
| REACTOME_SLC_MEDIATED_TRANSMEMBRANE_TRANSPORT | 240 | -0.3110831 | -1.4241834 | 0.00665779 | 0.139332 | 1 |
| REACTOME_AMINE_LIGAND_BINDING_RECEPTORS | 40 | -0.4118743 | -1.4225583 | 0.04095563 | 0.14047477 | 1 |
| REACTOME_ANTI_INFLAMMATORY_RESPONSE_FAVOURING_LEISHMANIA_PARASITE_INFECTION | 82 | -0.3508721 | -1.4146497 | 0.0237037 | 0.1461165 | 1 |
| REACTOME_IRE1ALPHA_ACTIVATES_CHAPERONES | 46 | -0.3908047 | -1.410748 | 0.04952077 | 0.14884634 | 1 |
| REACTOME_ANTIMICROBIAL_PEPTIDES | 73 | -0.3608587 | -1.4097579 | 0.0266876 | 0.14941937 | 1 |
| REACTOME_FOXO_MEDIATED_TRANSCRIPTION | 65 | -0.36537 | -1.4020605 | 0.03869969 | 0.1554271 | 1 |
| REACTOME_DDX58_IFIH1_MEDIATED_INDUCTION_OF_INTERFERON_ALPHA_BETA | 78 | -0.3508556 | -1.4013609 | 0.02647975 | 0.15571004 | 1 |
| REACTOME_CARGO_RECOGNITION_FOR_CLATHRIN_MEDIATED_ENDOCYTOSIS | 101 | -0.3351317 | -1.4000001 | 0.03303303 | 0.15685645 | 1 |
| KEGG_FC_GAMMA_R_MEDIATED_PHAGOCYTOSIS | 89 | -0.3466623 | -1.3991808 | 0.04081633 | 0.15732178 | 1 |
| REACTOME_CLATHRIN_MEDIATED_ENDOCYTOSIS | 141 | -0.3232346 | -1.3983041 | 0.02725969 | 0.15765908 | 1 |
| REACTOME_CIRCADIAN_CLOCK | 65 | -0.3616553 | -1.3969054 | 0.04545455 | 0.15839872 | 1 |
| REACTOME_AUTOPHAGY | 131 | -0.3229164 | -1.3937607 | 0.02898551 | 0.16117047 | 1 |
| KEGG_COMPLEMENT_AND_COAGULATION_CASCADES | 66 | -0.3571344 | -1.3933076 | 0.03376206 | 0.16138661 | 1 |
| REACTOME_FCGAMMA_RECEPTOR_FCGR_DEPENDENT_PHAGOCYTOSIS | 91 | -0.3424873 | -1.3919545 | 0.0309119 | 0.16257054 | 1 |
| REACTOME_PI3K_AKT_SIGNALING_IN_CANCER | 103 | -0.3356299 | -1.3900852 | 0.02276176 | 0.16373336 | 1 |
| KEGG_FC_EPSILON_RI_SIGNALING_PATHWAY | 75 | -0.3531322 | -1.3894926 | 0.04031008 | 0.16409247 | 1 |
| REACTOME_POST_TRANSLATIONAL_MODIFICATION_SYNTHESIS_OF_GPI_ANCHORED_PROTEINS | 81 | -0.3469557 | -1.3864834 | 0.03692308 | 0.16618565 | 1 |
| REACTOME_G_ALPHA_S_SIGNALLING_EVENTS | 149 | -0.3131306 | -1.3750393 | 0.02064897 | 0.17595088 | 1 |
| REACTOME_EXTRACELLULAR_MATRIX_ORGANIZATION | 285 | -0.2843373 | -1.3393153 | 0.01752022 | 0.2054292 | 1 |
| REACTOME_REGULATION_OF_INSULIN_LIKE_GROWTH_FACTOR_IGF_TRANSPORT_AND_UPTAKE_BY_INSULIN_LIKE_GROWTH_FACTOR_BINDING_PROTEINS_IGFBPS | 118 | -0.3076372 | -1.3193876 | 0.04852941 | 0.22306833 | 1 |

**Supplementary Table 13.** Biological processes enriched in the Non-Responders (Mandard Score 1-2) relative to Responders (Mandard Score 3-5) to neo-adjuvant therapy following Gene Set Enrichment Analysis with the KEGG and REACTOME gene sets (limited to nominal p value <0.05, FDR <0.2).

| NAME | SIZE | ES | NES | NOM p-val | FDR q-val | FWER p-val |
| --- | --- | --- | --- | --- | --- | --- |
| REACTOME_PROCESSING_OF_CAPPED_INTRON_CONTAINING_PRE_MRNA | 268 | 0.41249856 | 2.1323333 | 0 | 0.00925774 | 0.064 |
| REACTOME_RRNA_MODIFICATION_IN_THE_NUCLEUS_AND_CYTOSOL | 52 | 0.5411895 | 2.162976 | 0 | 0.00953518 | 0.044 |
| REACTOME_MRNA_SPLICING | 202 | 0.43570495 | 2.16921 | 0 | 0.01214163 | 0.042 |
| REACTOME_RNA_POLYMERASE_II_TRANSCRIPTION_TERMINATION | 67 | 0.47176427 | 2.003333 | 0 | 0.03320866 | 0.346 |
| REACTOME_EPIGENETIC_REGULATION_OF_GENE_EXPRESSION | 84 | 0.43039742 | 1.8770179 | 0 | 0.07528398 | 0.855 |
| REACTOME_TRANSCRIPTION_OF_E2F_TARGETS_UNDER_NEGATIVE_CONTROL_BY_DREAM_COMPLEX | 19 | 0.5882661 | 1.8582436 | 0.00241546 | 0.07762431 | 0.907 |
| REACTOME_TRNA_PROCESSING_IN_THE_NUCLEUS | 52 | 0.46107402 | 1.8258877 | 0 | 0.08900148 | 0.951 |
| REACTOME_TRNA_PROCESSING | 93 | 0.4030537 | 1.8075864 | 0 | 0.09107392 | 0.974 |
| KEGG_STARCH_AND_SUCROSE_METABOLISM | 35 | 0.48383203 | 1.7846048 | 0 | 0.09187856 | 0.986 |
| REACTOME_SIGNALING_BY_NTRK2_TRKB | 24 | 0.5530488 | 1.7896125 | 0.0046083 | 0.0920286 | 0.984 |
| REACTOME_BUTYRATE_RESPONSE_FACTOR_1_BRF1_BINDS_AND_DESTABILIZES_MRNA | 17 | 0.600394 | 1.7780842 | 0.00696056 | 0.09344514 | 0.99 |
| REACTOME_SYNTHESIS_OF_GLYCOSYLPHOSPHATIDYLINOSITOL_GPI | 18 | 0.5846689 | 1.7648596 | 0.00705882 | 0.10165159 | 0.994 |
| KEGG_SPLICEOSOME | 120 | 0.3690558 | 1.729121 | 0 | 0.12467326 | 1 |
| KEGG_PENTOSE_AND_GLUCURONATE_INTERCONVERSIONS | 17 | 0.5732097 | 1.711642 | 0.01650943 | 0.13728961 | 1 |
| KEGG_AMINOACYL_TRNA_BIOSYNTHESIS | 22 | 0.5146541 | 1.6991311 | 0.00909091 | 0.14270504 | 1 |
| REACTOME_G2_M_CHECKPOINTS | 124 | 0.35811177 | 1.7007219 | 0 | 0.14547433 | 1 |
| REACTOME_SIGNALING_BY_PDGFR_IN_DISEASE | 20 | 0.53356767 | 1.6818768 | 0.01891253 | 0.1518425 | 1 |
| REACTOME_TRANSCRIPTION_OF_E2F_TARGETS_UNDER_NEGATIVE_CONTROL_BY_P107_RBL1_AND_P130_RBL2_IN_COMPLEX_WITH_HDAC1 | 16 | 0.5583412 | 1.6528832 | 0.02905569 | 0.15259802 | 1 |
| REACTOME_MITOTIC_PROMETAPHASE | 181 | 0.34204012 | 1.6831865 | 0 | 0.1526461 | 1 |
| REACTOME_PROCESSING_OF_INTRONLESS_PRE_MRNAS | 20 | 0.5394347 | 1.685107 | 0.01481482 | 0.15283938 | 1 |
| REACTOME_HATS_ACETYLATE_HISTONES | 70 | 0.38460037 | 1.6499851 | 0 | 0.15446828 | 1 |
| REACTOME_RESOLUTION_OF_D_LOOP_STRUCTURES | 30 | 0.47807902 | 1.6760638 | 0.01851852 | 0.15485258 | 1 |
| REACTOME_RHOV_GTPASE_CYCLE | 36 | 0.4559446 | 1.68541 | 0.01234568 | 0.15488859 | 1 |
| REACTOME_RRNA_PROCESSING | 174 | 0.3396232 | 1.6553836 | 0 | 0.15535434 | 1 |
| REACTOME_CELL_CYCLE_CHECKPOINTS | 235 | 0.32445073 | 1.6476241 | 0 | 0.15551932 | 1 |
| REACTOME_KSRP_KHSRP_BINDS_AND_DESTABILIZES_MRNA | 17 | 0.5532095 | 1.6736476 | 0.01609195 | 0.15586011 | 1 |
| REACTOME_M_PHASE | 321 | 0.31445867 | 1.6575965 | 0 | 0.15674868 | 1 |
| REACTOME_TRNA_AMINOACYLATION | 24 | 0.5068883 | 1.6653268 | 0.01213592 | 0.1574205 | 1 |
| REACTOME_TRANSPORT_OF_MATURE_TRANSCRIPT_TO_CYTOPLASM | 79 | 0.38760814 | 1.6698315 | 0 | 0.15833677 | 1 |
| REACTOME_POLO_LIKE_KINASE_MEDIATED_EVENTS | 16 | 0.572613 | 1.6661453 | 0.01360544 | 0.158434 | 1 |
| REACTOME_TRISTETRAPROLIN_TTP_ZFP36_BINDS_AND_DESTABILIZES_MRNA | 17 | 0.55237633 | 1.659088 | 0.02059497 | 0.15909122 | 1 |
| REACTOME_PROCESSING_OF_CAPPED_INTRONLESS_PRE_MRNA | 29 | 0.47776002 | 1.6598485 | 0.01438849 | 0.16024189 | 1 |
| REACTOME_MRNA_SPLICING_MINOR_PATHWAY | 48 | 0.42279243 | 1.6399745 | 0.00726392 | 0.16129123 | 1 |
| REACTOME_NUCLEAR_PORE_COMPLEX_NPC_DISASSEMBLY | 32 | 0.46145305 | 1.6194974 | 0.01421801 | 0.17204939 | 1 |
| REACTOME_CELL_CYCLE_MITOTIC | 463 | 0.29415846 | 1.6137483 | 0 | 0.17402785 | 1 |
| REACTOME_SIGNALING_BY_NTRK3_TRKC | 16 | 0.5558653 | 1.6079406 | 0.02455357 | 0.17666717 | 1 |
| REACTOME_MITOCHONDRIAL_TRANSLATION | 91 | 0.35745183 | 1.5970837 | 0.00268817 | 0.1844066 | 1 |
| REACTOME_SIGNALING_BY_ERBB2_ECD_MUTANTS | 15 | 0.5336779 | 1.5778497 | 0.03512881 | 0.19857888 | 1 |
| REACTOME_SUMOYLATION_OF_DNA_REPLICATION_PROTEINS | 42 | 0.4201528 | 1.5783302 | 0.01058201 | 0.19961198 | 1 |
| REACTOME_REGULATION_OF_PLK1_ACTIVITY_AT_G2_M_TRANSITION | 82 | 0.361365 | 1.5690627 | 0.00277008 | 0.20300116 | 1 |
| REACTOME_G1_S_SPECIFIC_TRANSCRIPTION | 29 | 0.4532493 | 1.5611504 | 0.03722084 | 0.20614123 | 1 |
| REACTOME_REGULATION_OF_RAS_BY_GAPS | 64 | 0.37740186 | 1.558177 | 0.02067184 | 0.20911974 | 1 |
| REACTOME_RECRUITMENT_OF_MITOTIC_CENTROSOME_PROTEINS_AND_COMPLEXES | 76 | 0.36227074 | 1.5443127 | 0.02754821 | 0.21961817 | 1 |
| KEGG_RNA_DEGRADATION | 50 | 0.39730138 | 1.5357333 | 0.0175 | 0.22187822 | 1 |
| REACTOME_MITOCHONDRIAL_TRNA_AMINOACYLATION | 18 | 0.5029831 | 1.532276 | 0.03381642 | 0.22553307 | 1 |
| REACTOME_SNRNP_ASSEMBLY | 48 | 0.3893176 | 1.5303276 | 0.01190476 | 0.22569084 | 1 |
| REACTOME_PKMTS_METHYLATE_HISTONE_LYSINES | 34 | 0.42312565 | 1.5207014 | 0.02147971 | 0.23509222 | 1 |
| REACTOME_ACTIVATION_OF_THE_PRE_REPLICATIVE_COMPLEX | 33 | 0.42477077 | 1.5169983 | 0.02552204 | 0.23645386 | 1 |
| REACTOME_MITOTIC_PROPHASE | 77 | 0.3508161 | 1.5104983 | 0.01369863 | 0.23808263 | 1 |
| REACTOME_TRANSCRIPTIONAL_REGULATION_BY_E2F6 | 34 | 0.41925016 | 1.5093473 | 0.04098361 | 0.23847574 | 1 |
| REACTOME_REGULATION_OF_MRNA_STABILITY_BY_PROTEINS_THAT_BIND_AU_RICH_ELEMENTS | 83 | 0.3456682 | 1.5109965 | 0.0109589 | 0.23871014 | 1 |
| REACTOME_DNA_REPLICATION_PRE_INITIATION | 94 | 0.3357974 | 1.5115824 | 0.01630435 | 0.23934105 | 1 |
| REACTOME_NEGATIVE_EPIGENETIC_REGULATION_OF_RRNA_EXPRESSION | 45 | 0.39963627 | 1.512078 | 0.02083333 | 0.24003004 | 1 |
| REACTOME_TRANSPORT_OF_THE_SLBP_DEPENDANT_MATURE_MRNA | 32 | 0.418895 | 1.503623 | 0.04438642 | 0.24216563 | 1 |
| REACTOME_TRAFFICKING_OF_GLUR2_CONTAINING_AMPA_RECEPTORS | 17 | 0.5018108 | 1.49514 | 0.04794521 | 0.2486404 | 1 |

**Supplementary Table 14**. Differentially expressed genes between responders (n=3) and non-responders (n=23) to neoadjuvant chemotherapy from SAMR analysis of respective patient biopsy samples (FDR q-value <0.2/-log10 q-value > 0.6), where ‘increased’ indicates genes significantly upregulated in non-responders and ‘decreased’ indicates genes significantly upregulated in responders to neoadjuvant treatment.

| **Gene Name** | **Change** | **Fold change (log2)** | **-LOG10(q-value)** | **q-value** |
| --- | --- | --- | --- | --- |
| CXCL5 | Decreased | -4.22 | 0.97502074 | 0.106 |
| TREML3 | Decreased | -4.18 | 2 | 0.010 |
| BCL2A1 | Decreased | -3.19 | 0.97502074 | 0.106 |
| ALOX5AP | Decreased | -1.97 | 0.97502074 | 0.106 |
| NFIL3 | Decreased | -1.77 | 2 | 0.010 |
| VDAC1P4 | Decreased | -1.63 | 2 | 0.010 |
| FPR1 | Decreased | -1.62 | 0.702019468 | 0.199 |
| CD83 | Decreased | -1.55 | 0.97502074 | 0.106 |
| CSF2RB | Decreased | -1.51 | 0.97502074 | 0.106 |
| TNFRSF9 | Decreased | -1.32 | 0.702019468 | 0.199 |
| SNAI1 | Decreased | -1.17 | 2 | 0.010 |
| METTL16 /// --- | Decreased | -1.07 | 2 | 0.010 |
| MIR22HG | Increased | 2.17 | 0.702019468 | 0.199 |
| GTF3C2 | Increased | 1.73 | 1.016413424 | 0.096 |
| C22orf46 /// --- | Increased | 1.38 | 0.702019468 | 0.199 |
