# Supplementary material for: Immune microenvironment modulation following neoadjuvant therapy for oesophageal adenocarcinoma: a translational analysis of the DEBIOC clinical trial
Source: ESMO Open. 2024 Oct 11;9(11):103930. doi: 10.1016/j.esmoop.2024.103930 (PMC11693431; doi:10.1016/j.esmoop.2024.103930)
Supplement: Supplementary Methods [file mmc2.docx]

**Supplementary Methods for ‘Immune Microenvironment Modulation following Neo-adjuvant Therapy for Oesophageal Adenocarcinoma: A Translational Analysis of the DEBIOC Clinical Trial’**

All data analysis was performed using R statistical software unless otherwise stated.^1^

**Transcriptomic Profiling**

Transcriptomic profiling of 25 pre-treatment FFPE OAC endoscopic biopsies and 18 surgical resection specimens was performed using the Almac Diagnostics Xcel™ array (Almac, Craigavon, UK). All specimens underwent pathological review before marking for microdissection, with samples containing >30% tumour content included in the analysis. Total RNA was extracted from 7X 10uM sections using the Roche High Pure RNA Paraffin Kit for FFPE (F Hoffman-La-Roche, Basel, Switzerland) and amplified using NuGen Ovation FFPE Amplification System v3 (NuGen San Carlos, CA). The amplified product was hybridized to the Almac Xcel™ array (Almac Diagnostic Services, Craigavon, UK), which is a cDNA microarray-based technology optimised for archival FFPE tissue, and analysed using the Affymetrix 7G scanner (Affymetrix, Santa Clara, CA).

**Almac Diagnostics’ clara^T^ Total mRNA Report**

Samples which passed quality control were processed with the Almac Diagnostic Services proprietary analysis pipeline and reporting software using version 3.0.0 of the clara^T^ Total mRNA report (Almac Diagnostic Services, <https://www.almacgroup.com/diagnostics/claratreport/>). Clara^T^ provides a comprehensive overview of tumour profiles using 92 gene signatures, 100 unique single gene drug targets (SGDTs) and 7337 single genes across the 10 hallmarks of cancer (Supplementary Figure 3). Content includes Almac proprietary assays and public signatures, all of which are published and biologically relevant in either pre-clinical or clinical sample cohorts. Outputs of the reported samples were signature scores and single gene drug target expression with values indicated by the continuous expression of each within the sample profile relative to the tested cohort represented by percentile ranks allowing comparison of signature score changes across and between samples and phenotypes. A previously published, pre-defined threshold of 0.3403 was used to define DNA Damage Immune Response (DDIR) signature status, whereby a score ﻿>0.3403 was classified as ﻿DDIR positive and ≤0.3403 as DDIR negative.^21^

**Comparison of Signature Scores Pre and Post Neoadjuvant Chemotherapy**

To investigate significant changes in gene signature scores pre- and post- assigned neoadjuvant chemotherapy, paired patient biopsy and resection samples only (n=17) were used to assess changes from baseline in each treatment arm (Xelox Only n=6; Xelox plus AZD8931 n=11), and were summarised by percentage of signatures showing significant change per hallmark. For this analysis, raw signature scores were assessed with Wilcoxon Signed Rank Test and correction for multiple testing carried out using the False Discovery Rate (FDR) Benjamini-Hochberg (BH) method with p-values deemed significant at <0.2.

**Correlation Analysis Between Immune Response, EMT and Angiogenesis**

To note any significant correlations between gene signatures representing response to immune checkpoint blockade, resistance to immune checkpoint blockade, angiogenesis and EMT correlation plots were constructed using the “Hmisc” (available: [https://CRAN.R-project.org/package=Hmisc](https://cran.r-project.org/package=Hmisc)) and “corrplot” (available: <https://github.com/taiyun/corrplot>) packages in R.^2,3^ Non-parametric Spearman’s Rank correlation was carried out amongst the chosen signatures, plotting only significant values at p<0.05 in heatmaps. Correlations were carried out between treatment types, biopsy and resection specimens to determine significant differences between phenotypes assessed.

**Pathway Enrichment Analysis of Responder and Non-Responder Biopsies Using Gene Expression Data**

To investigate biological differences between (n=3) responders and (n=22) non-responders to treatment outside of the hallmarks of cancer and their corresponding gene signature scores from clara^T^, differential gene expression analysis was carried out on 21,384 genes between response phenotypes based on gene expression profiles at biopsy using SAMR (accessible: <https://CRAN.R-project.org/package=samr>).^4^ A pre-ranked gene list generated from SAMR was utilised based on fold change between comparative groups as input to Gene Set Enrichment Analysis (GSEA) (available at: <https://www.gsea-msigdb.org/gsea/>), to reveal significantly enriched signalling pathways between phenotypes at biopsy utilising the MSigDB C2-all signature database as a gene set reference.^5,6^ Results were then filtered to only those with a nominal p-value <0.05, and FDR q-value <0.2, in addition to only those pathways resulting from KEGG or Reactome databases. The top results ranked by FDR q-value and Normalised Enrichment Score (NES) where then plotted to highlight enriched pathways per phenotype. A volcano plot was created using ‘VolcaNoseR’ (accessible at: <https://huygens.science.uva.nl/VolcaNoseR/>) to highlight the differentially expressed genes per phenotype resulting from SAMR based on FDR q-value <0.2, and log2 Fold change either < -1 for responders or > 1 for non-responder phenotypes.^7^

**Visualisation of Immune Subtype Changes from Biopsy to Resection Resulting from Clustering Analysis**

Immune subtypes derived from clustering of biopsy samples were visualised in a heatmap along with clinical factors of significance determined by Fisher’s Exact testing. The heatmap was constructed of multiple panels consisting of gene expression signature scores (percentile ranks) representing acquired immune signalling, innate immune signalling, interferon signalling, single gene drug targets representing immune checkpoints, signatures relating to immune checkpoint blockade (ICB) response, signatures relating to ICB resistance in addition to signatures representative of angiogenesis and EMT.

To visualise notable signalling changes in the biopsy derived immune subtypes from pre- to post- neoadjuvant treatment, heatmaps of resection samples were constructed from patient matched resection samples where possible (n=17 matched resection samples). E.g., If patient 1 has a biopsy assigned to the immune mixed cluster in the biopsy heatmap, then patient 1’s matched resection would be in the immune mixed cluster in the resection heatmap, showing the same hallmark signatures noted from the biopsy heatmap, yet with signature scores reflective of the resection. This analysis allowed for the visualisation of immune signalling changes occurring post-treatment.

**EGFR Fluorescent In Situ Hybridisation (FISH)**

EGFR FISH was performed and scored using an established protocol in NHS Grampian.^23^ Briefly, 4μm sections were mounted, baked at 50^o^C for 2 hours, deparaffinised and processed using the Cytocell Aquarius® kit. Vysis EGFR/CEP7 dual colour probe (Abbott Laboratories, Maidenhead, UK) was applied followed by hybridisation at 37^o^C for 16 hours overnight and a nuclear counter stain containing 4’,6-diamidino-2-phenylindole (DAPI) (Vectashield mounting medium, Vector Laboratories, Peterborough, UK) was added. EGFR copy number gain was classified using a 6-point scale. Tumours scoring 5 (high polysomy) or 6 (amplification) were classified as EGFR FISH positive; tumours scoring 1 to 4 were defined as EGFR FISH negative.^23^ Analysis was performed by one scorer, with any samples with a borderline result leading to further analysis by a second independent scorer.

**Identification and Quantification of TILs**

For our digital assessment of tumour infiltrating lymphocytes (TILs), we obtained digitally scanned Haematoxylin and Eosin (H&E) whole-slide images consisting of pre-treatment biopsies and surgical resection specimens from matched patients where possible. To perform our immune assessment, we employed QuPath, an open-source image analysis software.^8^ Each image was imported into the software and labelled according to the patient trial number, the type of staining (H&E) and the source of the image (biopsy vs surgery). Within QuPath, we utilized the built-in colour deconvolution method to separate the basic, fundamental stains in all the H&E images. Subsequently, we viewed each slide individually at low magnifications first (x5 and x10), and then at higher magnifications (x20 and x40) to create regions of interest (ROIs) that encase the tumour and its stromal component. The selection and assessment of ROIs adhered to the established guidelines and were reviewed prior to immune analysis with a consultant pathologist (JAJ).^9^ The ROIs typically included all epithelial component of the tumour and only its adjacent stromal component, excluding areas of necrosis, artefacts such as folds or crush artefact and native lymphoid tissues if present. Notably, for two patients, “surgery” images showed complete response, and therefore, the ROI enclosed an area containing the tumour bed post-surgical removal. After assigning the ROIs, we applied QuPath’s built-in Cell Detection method to detect and segment each cell in the ROI. The parameters for the cell detection were normalised for all images to maintain consistency. Following cell detection, we classified cells as “immune cell” or “non-immune cell” using handcrafted features within QuPath. After extracting all classified cell detection measurements into an excel sheet, the percentage of the of immune cells counts in the ROI was calculated by dividing the number of cells classified as “immune cell” by the number of “total detections” in the ROI.

**Supplementary Methods References**

1. R Core Team (2023). R: A language and environment for statistical computing. R Foundation for Statistical Computing, Vienna, Austria. URL <https://www/R-project.org/>.

2. Harrell Jr F (2023). _Hmisc: Harrell Miscellaneous_. R package version 5.1-1, <https://CRAN.R-project.org/package=Hmisc>.

3. Taiyun Wei and Viliam Simko (2021). R package 'corrplot': Visualization of a Correlation Matrix (Version 0.92). Available from <https://github.com/taiyun/corrplot>.

4. Tibshirani R, Seo MJ, Chu G, Narasimhan B, Li J (2018). _samr: SAM: Significance Analysis of Microarrays_. R package version 3.0, <https://CRAN.R-project.org/package=samr>.

5. Subramanian A, Tamayo P, Mootha VK, Mukherjee S, Ebert BL, Gillette MA, Paulovich A, Pomeroy SL, Golub TR, Lander ES, Mesirov JP. Gene set enrichment analysis: a knowledge-based approach for interpreting genome-wide expression profiles. Proc Natl Acad Sci U S A. 2005 Oct 25;102(43):15545-50. doi: 10.1073/pnas.0506580102. Epub 2005 Sep 30. PMID: 16199517; PMCID: PMC1239896.

6. Mootha VK, Lindgren CM, Eriksson KF, Subramanian A, Sihag S, Lehar J, Puigserver P, Carlsson E, Ridderstråle M, Laurila E, Houstis N, Daly MJ, Patterson N, Mesirov JP, Golub TR, Tamayo P, Spiegelman B, Lander ES, Hirschhorn JN, Altshuler D, Groop LC. PGC-1alpha-responsive genes involved in oxidative phosphorylation are coordinately downregulated in human diabetes. Nat Genet. 2003 Jul;34(3):267-73. doi: 10.1038/ng1180. PMID: 12808457.

7. Goedhart J, Luijsterburg MS. VolcaNoseR is a web app for creating, exploring, labeling and sharing volcano plots. Sci Rep. 2020 Nov 25;10(1):20560. doi: 10.1038/s41598-020-76603-3. PMID: 33239692; PMCID: PMC7689420.

8. Bankhead P, Loughrey MB, Fernández JA, Dombrowski Y, McArt DG, Dunne PD, et al. QuPath: Open source software for digital pathology image analysis. Sci Rep. 2017;7(1):1–7.

9. Hendry S, Salgado R, Gevaert T, Russell PA, John T, Thapa B, et al. Assessing Tumor-infiltrating Lymphocytes in Solid Tumors: A Practical Review for Pathologists and Proposal for a Standardized Method from the International Immunooncology Biomarkers Working Group: Part 1: Assessing the Host Immune Response, TILs in Invasi. Adv Anat Pathol. 2017;24(5):235–51.
